# Target Specific Inhibition of Protein Tyrosine Kinase in Conjunction With Cancer and SARS-COV-2 by Olive Nutraceuticals

**DOI:** 10.3389/fphar.2021.812565

**Published:** 2022-03-08

**Authors:** Arabinda Ghosh, Nobendu Mukerjee, Bhavdeep Sharma, Anushree Pant, Yugal Kishore Mohanta, Rahul D. Jawarkar, Ravindrakumar L. Bakal, Ermias Mergia Terefe, Gaber El-Saber Batiha, Gomaa Mostafa-Hedeab, Nisreen Khalid Aref Albezrah, Abhijit Dey, Debabrat Baishya

**Affiliations:** ^1^ Microbiology Division, Department of Botany, Gauhati University, Guwahati, India; ^2^ Department of Microbiology, Ramakrishna Mission Vivekananda Centenary College, Kolkata, India; ^3^ Department of Biotechnology, Thapar Institute of Engineering and Technology, Patiala, India; ^4^ Department of Applied Biology, University of Science and Technology Meghalaya, Ri Bhoi, India; ^5^ Department of Medicinal Chemistry, Dr. Rajendra Gode Institute of Pharmacy, Amravati, India; ^6^ Department of Pharmacology and Pharmacognosy, School of Pharmacy and Health Sciences, United states International University-Africa, Nairobi, Kenya; ^7^ Department of Pharmacology and Therapeutics, Faculty of Veterinary Medicine, Damanhour University, Damanhour, Egypt; ^8^ Pharmacology Department & Health Research Unit–Medical College–Jouf University, Sakakah, Saudi Arabia; ^9^ Pharmacology Department, Faculty of Medicine, Beni-Suef University, Beni-Suef, Egypt; ^10^ Obstetrics and Gynecology Department, College of Medicine, Taif University, Taif, Saudi Arabia; ^11^ Department of Life Sciences, Presidency University, Kolkata, India; ^12^ Department of Bioengineering, Gauhati University, Guwahati, India

**Keywords:** SARS CoV-2, ALK, BTK, Wedelosin, Olive, MD simulation, QSAR

## Abstract

The fact that viruses cause human cancer dates back to the early 1980s. By reprogramming cellular signaling pathways, viruses encoded protein that can regulate altered control of cell cycle events. Viruses can interact with a superfamily of membrane bound protein, receptor tyrosine kinase to modulate their activity in order to increase virus entrance into cells and promotion of viral replication within the host. Therefore, our study aimed at screening of inhibitors of tyrosine kinase using natural compounds from olive. Protein tyrosine kinase (PTK) is an important factor for cancer progression and can be linked to coronavirus. It is evident that over expression of Protein tyrosine kinase (PTK) enhance viral endocytosis and proliferation and the use of tyrosine kinase inhibitors reduced the period of infection period. Functional network studies were carried out using two major PTKs viz. Anaplastic lymphoma kinase (ALK) and B-lymphocytic kinase (BTK). They are associated with coronavirus in regulation of cell signaling proteins for cellular processes. We virtually screened for 161 library of natural compounds from olive found overexpressed in ALK and BTK in metastatic as well as virus host cells. We have employed both ligand and target-based approach for drug designing by high throughput screening using Multilinear regression model based QSAR and docking. The QSAR based virtual screening of 161 olive nutraceutical compounds has successfully identified certain new hit; Wedelosin, in which, the descriptor rsa (ratio of molecular surface area to the solvent accessible surface area) plays crucial role in deciding Wedelosin’s inhibitory potency. The best-docked olive nutraceuticals further investigated for the stability and effectivity of the BTK and ALK during in 150 ns molecular dynamics and simulation. Post simulation analysis and binding energy estimation in MMGBSA further revealed the intensive potential of the olive nutraceuticals in PTK inhibition. This study is therefore expected to widen the use of nutraceuticals from olive in cancer as well as SARS-CoV2 alternative therapy.

## Graphical Abstract

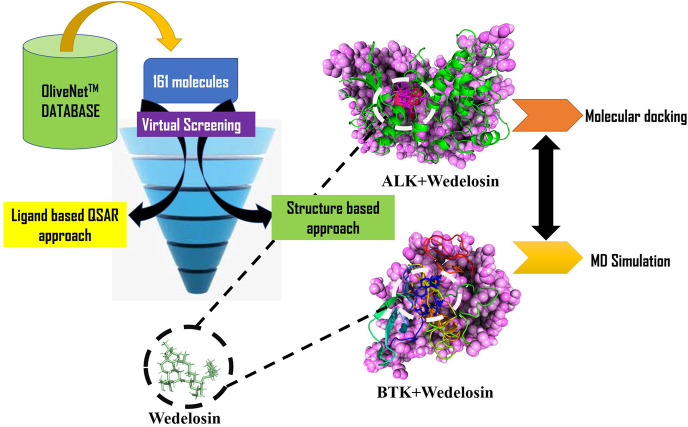

## Introduction

Following the pandemic SARS-CoV-2 infection, an increasing number of coronavirus disease-19 (COVID-19) cases have been reported globally since December 2019. COVID-19 currently lacks a specific treatment for SARS and associated multiorgan failure. Liang and colleagues recently reported an elevated likelihood of COVID-19 in cancer patients, which is linked to a worse COVID-19 prognosis. Thus, worldwide bodies have advised delaying or suspending anticancer therapies, raising concerns about cancer progression. Targeted tyrosine kinase inhibitors (TKI) are highly effective in treating oncogene-dependent non-small cell lung cancer (NSCLC) (TKIs). Withdrawal of TKIs may be harmful to this patient subgroup. This is especially true when SARS-CoV-2 lung infection is discovered in asymptomatic patients. We report two examples of oncogene-driven NSCLC patients infected by SARS-CoV-2 who maintained targeted therapy with ALK/ROS1 TKIs and recovered without special antiviral therapies (Leonetti et al., 2020). In SARS-CoV-2 pandemic the Growth factor receptors (GFRs) with intrinsic protein kinase activity are in much consideration. GFRs are relevant for the entry of multiple viruses, including coronaviruses, which makes them a central topic of discussion regarding the SARS-CoV-2 pandemic (Purcaru et al., 2021).

GFRs auto-phosphorylates at *Tyr* residues by interacting with extracellular growth factors (Carapancea et al., 2009). This auto-phosphorylation causes cascade of reactions in various downstream signalling pathways like JAK/STAT, Ras/ERK/MAPK, which regulates the cell growth, metabolism and differentiation (Schlessinger, 2000). Dysregulation of the downstream signaling pathways may lead to cancer progression, hence protein tyrosine kinase inhibitors are targeted to GFR in most anticancer therapies. The epidermal growth factor receptor (EGFR) belongs to ErbB family of protein tyrosine kinase receptors, and aids the internalization of the viruses, hence can be a potential entry point for coronavirus (Schlessinger, 2014). The over expression of the EGFR in human cancers is well documented. For years the tyrosine kinase inhibitors are used in clinical studies to target the EGFR for the treatment of cancers like of non-small-cell lung cancer (Liu et al., 2017). A recent study has reported the overactive EGFR signalling in the lung tissue along with increased level of inflammation following SARS-CoV infection (Venkataraman et al., 2017).

A well-known PTK associated with EGFR is Anaplastic lymphoma kinase (ALK) was identified in lymphoma, non-small cell lung cancer is the most common ALK-positive disease. It aids in the growth of the intestines and the nervous system. Anaplastic lymphoma kinase (ALK), also known as CD246 or Anaplastic lymphoma kinase (ALK). When ALK is expressed in spitz tumours, the tumours have a typical amelanotic look, and their development pattern is characterized by crossing fascicles. Activated ALK is a transmembrane tyrosine kinase receptor that dimerizes and auto phosphorylates the intracellular kinase domain upon ligand interaction (Della et al., 2018). The tyrosine kinase receptor anaplastic lymphoma kinase (ALK) has been linked to the development of many tumours. Clinical studies on another PTK, Bruton tyrosine kinase (BTK) inhibition revealed the ameliorating cancer in SARS-CoV-2 patients (Kifle, 2021). Again, for the treatment of patients with chronic lymphocytic leukaemia (CLL) and haematological malignancies (HM), BTK inhibitor like ibrutinib is the most advanced in clinical development (Aalipour and Advani, 2014; Wang et al., 2015). HM is associated with BTK dysregulation, and COVID-19 mortality has been found to be higher in HM patients (Campbell et al., 2018; Pinato et al., 2020). These findings show a direct relationship between protein tyrosine kinase dysregulation and the severity of both cancer and COVID-19. Furthermore, cytotoxic therapy for cancer treatment has side effects such as leukopenia, which increases the patient’s risk of infection in the COVID-19 pandemic, complicating cancer treatment (Anil et al., 2020). Therefore, interests have been gained in discovering natural kinase inhibitors that can be used to treat both cancer and COVID-19.

Polyphenols, anthraquinones, alkaloids, and other phytochemicals act as protein tyrosine kinase inhibitors, and thus play an essential role in discovering and developing new potential drugs (Wang et al., 2014). The bioactive compounds epoxy-quino-phomopsins A and B from the endophytic fungus *Phomopsis* sp isolated from Moruscathayana demonstrated strong inhibitory properties against Bruton’s Tyrosine Kinase (nRTK) with their kinase activity (Hermawati et al., 2021). The computational analysis also revealed that compounds such as sulawesins A and B have comparable inhibition potential against Bruton’s tyrosine kinase (BTK) (Flamandita et al., 2020). Recent reports suggested that consuming olive polyphenols reduces the risk of developing cancer (Donaldson, 2004; Bach-Faig et al., 2011). Olive tree (*Olea europaea* L.) belongs to suborder Asteranae, order Lamiales, family Oleaceae, Genus Olea. Bioactive compounds derived from olives have been shown to have a wide range of bioactivities. Olive’s potential health benefits encourage the identification and characterization of bioactive compounds derived from it. The most comprehensive database, OliveNet™ is a valuable resource of compounds found in the various matrices of olives (Bonvino et al., 2018).

In this particular study, aim was to identify of novel compounds from olive OliveNet™ database having efficient activity against ALK and BTK kinases. Virtual screening was carried out on ligand based QSAR approach and structure-based docking approach were employed to find the best possible compound for efficient target (ALK and BTK) inhibition. Nevertheless, this study also encompassed the study of structural stability of target and best compound complex from olive with the help of molecular dynamics simulation studies. The overall strategy is to discover novel compound as potent inhibitor for ALK and BTK for future therapeutic purposes.

## Methodology

### Preparation of Protein and Ligand Molecules

The OliveNet™ database was used to obtain the library of ligands by screening the metabolites of *Olea europaea* (Bonvino et al., 2018). The smiles notation and the three-dimensional structures of the selected ligands were downloaded in SDF format from PubChem database (Kim et al., 2019), further ligand structure files were converted to PDB format using Open Babel software (O’Boyle et al., 2011). The energy minimization of the ligands was performed in UCSF Chimera software (Pettersen et al., 2004) using Amber ff 14 sb force field. The receptor used in the study are receptor tyrosine kinase, RCSB Protein Data Bank (PDB) (Berman et al., 2000) was used to download receptors Anaplastic lymphoma kinase (ALK) with (PDB id: 5FTO, Resolution: 2.22 Å) and Bruton’s tyrosine kinase (BTK) with (PDB id: 5J87, Resolution: 1.59 Å). The structures were chosen from the protein data bank due to the least missing residues while compared with the other available structures of ALK and BTK present in the database. The protein structure was prepared by removing ligand, water molecules, metal ions. Polar hydrogens were added and non-polar hydrogens were merged. Finally, Kollman charges were added to the protein molecule before converting to PDBQT format by AutoDock Tools (v.1.5.6) of the MGL software package (Forli et al., 2016).

### QSAR Modelling

#### Selection of Data-Set

**FIGURE 1:**
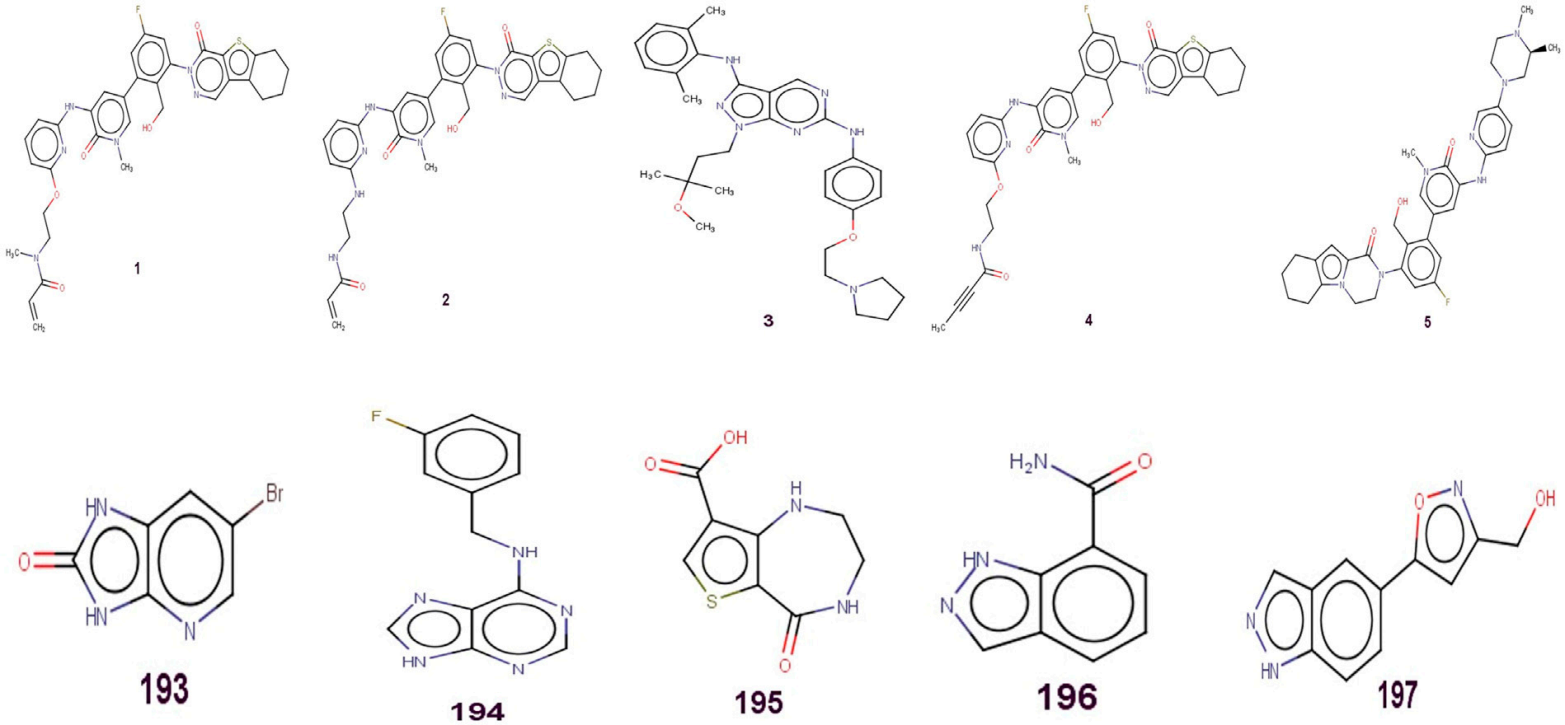
Presentation of Five least and five most active molecules.

A sequence of one hundred ninety-seven compounds, with stated enzyme inhibitory concentration (Ki) have been selected for the present work (Gilson et al., 2016). The Ki values extending from 0.146 to a hundred thousand nM had been converted to pKi (pKi = –logKi) earlier than genuine QSAR evaluation for the ease of managing of the data. Five least and five most active molecules are depicted in Figure 1 to show the variation in bio-activity with chemical features. Then, as a part of data curation, molecules with ambiguous enzyme inhibition constant (Ki) values, duplicates, salts, metal-based inhibitors, etc. were excluded (Consonni et al., 2009; Dearden et al., 2009; Huang and Fan, 2011; Gramatica et al., 2013; Cherkasov et al., 2014; Fujita and Winkler, 2016; Gramatica, 2020; Zaki et al., 2021). The SMILES strings with stated Ki and pKi values for all the molecules are presented in Supplementary Table S1 in the supplementary material.

#### Molecular Structure Drawing and Optimization

Drawing of 2D- Structures of all the one hundred and ninety seven molecules and their conversion to the corresponding 3D- structures was achieved using free and open source softwares ChemSketch 12 Freeware (www.acdlabs.com) and OpenBabel 2.4, respectively. Thereafter, force field MMFF94 available in TINKER (default settings) and Open3DAlign employed for an optimization and molecular alignment, respectively.

#### Molecular Descriptor Calculation and Objective Feature Selection (OFS)

The SMILES notations were converted to 3D-optimized structures the usage of Openbabel 3.1 before calculation of molecular descriptors (O’Boyle et al., 2011).

The success of a QSAR analysis considerably relies upon on appropriate calculation of diverse molecular descriptors to extend mechanistic interpretation, observed with the aid of their pruning to minimize the risk of overfitting from noisy redundant descriptors. To reap these goals, PyDescriptor was used to calculate greater than 30,000 molecular descriptors (Masand and Rastija 2017).

The huge pool of molecular descriptors includes 1D-to 3D-molecular descriptors. Then, OFS was once performed using QSARINS-2.2.4 to eliminate near constant, constant and quite inter-correlated (|R| > 0.90) molecular descriptors. The ultimate set is with 3,191 molecular descriptors, which nonetheless consists of manifold descriptors main to coverage of a large descriptor space (Gramatica et al., 2013).

#### Splitting the Data set Into Training and External sets and Subjective Feature Selection (SFS)

To avoid information leaking, prior to comprehensive subjective feature selection, the information set must be divided into training and prediction (also known as external or test set) sets with excellent composition and proportions (Masand et al., 2015).

The data set was separated into training (80% = 157 molecules) and prediction or external (20% = 40 molecules) sets at random to avoid bias. The main aim of a training set was to choose an acceptable number of molecular descriptors, whereas the prediction/external set was solely utilised to validate the model externally (Predictive QSAR). A genetic algorithm unified with multilinear regression (GA-MLR) technique created in QSARINS-2.2.4 was utilised to choose relevant descriptors utilising Q2LOO as a fitness parameter to select relevant descriptors. A sufficient number of molecular descriptors in the model is a critical step in creating a good QSAR model with no over-fitting and appropriate interpretability.

### Building Regression Model and its Validation

A good QSAR model that has been validated adequately using multiple methodologies such as cross-validation, external validation, Y-randomization, and applicability domain (Williams plot) is useful for future use in virtual screening, molecular optimization, and decision making, among other things. The statistical parameters listed below are typically used to validate a model, along with their recommended threshold values (Chirico and Gramatica, 2011; Chirico and Gramatica, 2011; Huang and Fan, 2011; Roy et al., 2011; Martin et at., 2012; Cherkasov et al., 2014; Krstajic et al., 2014; Consonni et al., 2019): *R* ^2^_tr_ ≥ 0.6, *Q* ^2^_loo_ ≥ 0.5, *Q* ^2^_LMO_ ≥ 0.6, *R* ^2^ > *Q* ^2^, *R* ^2^_ex_ ≥ 0.6, *RMSE*_*tr*_ < *RMSE*_*cv*_, ΔK ≥ 0.05, *CCC* ≥ 0.80, *Q* ^2^-*F* ^n^ ≥ 0.60, *r* ^2^_m_ ≥ 0.5, (1-*r* ^2^/*r*_o_^2^) < 0.1, 0.9 ≤ *k* ≤ 1.1 or (1-*r* ^2^/*r’*_o_^2^) < 0.1, 0.9 ≤ *k’* ≤ 1.1,| *r*_o_^2^− *r’*_o_^2^| < 0.3, *RMSE*_*ex,*_ *MAE*_*ex*_, *R* ^*2*^_*ex*_ *, Q* ^*2*^_*F1*_, *Q* ^*2*^_*F2*_, *Q* ^*2*^_*F3*_, and low *R* ^2^_Yscr_, *RMSE* and *MAE*. (The formulae for calculating these statistical parameters are available in Supplementary Table S1). In addition, Williams plot was plotted to evaluate the applicability domain of QSAR model.

### QSAR Based Virtual Screening

In QSAR based virtual screening, we have used 161 olive neutraceuticals. Accordingly, 161 were used for QSAR-based virtual screening. Erstwhile to molecular descriptor calculations, the 3D-structures of the molecules were arranged in the same way as modelling set. Then molecular descriptors were calculated for the 161 olive database compounds and the appropriately validated six parametric QSAR model was used to envisage the biological property of novel compounds.

### Virtual Screening of Natural Compounds Using Molecular Docking

The structure based virtual screening of the compounds was performed using autodock vina Ver 1.1.2 (Trott and Olson, 2010). Binding sites of BTK and ALK for screening were predicted using DoGSiteScorer (Volkamer et al., 2012) and information about the binding site of native ligand. The size of the grid box was set to be 96 × 52 × 56 Å for BTK and 78 × 76 × 58 Å for ALK, centered around identified binding site. The compounds with the best binding affinity (kcal/mol) corroborating ligand-based screening in QSAR analysis were selected for the studies.

### Molecular Docking for Validation of Docking Score

The best hit Wedelosin from the QSAR modelling and virtual screening were re-docked against Bruton’s tyrosine kinase (PDB id: 5J87) and Anaplastic lymphoma kinase (ALK) (PDB id: 5FTO). In order to validate the docking, crystal structures were docked with the native ligands entrectinib and N42 bound to X-ray structures of ALK and BTK, respectively. In addition to former, for comparison and better validation of docking at the binding cavity of ALK and BTK proteins the common approved drugs crizotinib and ibrutinib were also carried out, respectively. Protein and ligand preparation were done using AutoDock Tools (v.1.5.6) (Forli et al., 2016). Gasteiger charges were added to the ligand molecules prior converting to PDBQT format. Online server DoGSiteScorer and the information about the binding site residues of native ligand were used to construct the grid box. The grid box of dimensions 96 × 52 × 56 Å for BTK and 78 × 76 × 58 Å for ALK with 0.375 Å grid spacing was constructed using Autogrid 4.2. Semi flexible docking was done keeping the receptor molecule rigid and ligands flexible. Molecular docking was done via Autodock 4.2 (Morris et al., 2009) using the Lamarckian Genetic Algorithm (LGA) scoring function with number of GA runs = 100, population size = 500 and maximum number of evaluations = 25,000,000. After docking, the RMSD clustering maps were obtained by re-clustering command with a clustering tolerance 0.25 Å, 0.5 Å and 1 Å, respectively, in order to obtain the best cluster having lowest energy score with high number of populations.

### Molecular Dynamics Simulation (MD) and Free Energy Landscape Analysis

The MD simulations studies were carried in triplicate on dock complexes for ALK and BTK with Wedelosin using the Desmond 2020.1 from Schrödinger, LLC. The triplicate samplings were made using same parameters for each MD run in order to obtain reproducibility of the results. The OPLS-2005 force field (Bowers et al., 2006; Chow et al., 2008; Shivakumar et al., 2010) and explicit solvent model with the SPC water molecules were used in this system (Jorgensen et al., 1983). Na + ions were added to neutralize the charge 0.15 M, NaCl solutions were added to the system to simulate the physiological environment. Initially, the system was equilibrated using an NVT ensemble for 150 ns to retrain over the protein- Wedelosin complex. Following the previous step, a short run of equilibration and minimization was carried out using an NPT ensemble for 12 ns The NPT ensemble was set up using the Nose-Hoover chain coupling scheme (Martyna et al., 1994) with the temperature at 37°C, the relaxation time of 1.0 ps, and pressure 1 bar maintained in all the simulations. A time step of 2 fs was used. The Martyna-Tuckerman–Klein chain coupling scheme (Martyna et al., 1992) barostat method was used for pressure control with a relaxation time of 2 ps. The particle mesh Ewald method (Toukmaji and Board, 1996) was used for calculating long-range electrostatic interactions, and the radius for the coulomb interactions were fixed at 9 Å. RESPA integrator was used for a time step of 2 fs for each trajectory to calculate the bonded forces. The root means square deviation (RMSD), radius of gyration (Rg), root mean square fluctuation (RMSF) and number of hydrogen (H-bonds) and Solvent accessible surface area (SASA) were calculated to monitor the stability of the MD simulations. The free energy landscape of protein folding on Wedelosin bound complex was measured using Geo_measures v 0.8 (Kagami et al., 2020). Geo_measures include a powerful library of g_sham and form the MD trajectory against RMSD and radius of gyration (Rg) energy profile of folding recorded in a 3D plot using matplotlib python package.

### Molecular Mechanics Generalized Born and Surface Area (MMGBSA) Calculations

During MD simulations of ALK and BTK complexed with Wedelosin, the binding free energy (Gbind) of docked complexes was calculated using the premier molecular mechanics generalized Born surface area (MM-GBSA) module (Schrodinger suite, LLC, New York, NY, 2017-4). The binding free energy was calculated using the OPLS 2005 force field, VSGB solvent model, and rotamer search methods (Piao et al., 2019). After the MD run, 10 ns intervals were used to choose the MD trajectories frames. The total free energy binding was calculated using Eq. 1:

ΔGbind = Gcomplex - (Gprotein + Gligand) (1)

Where, ∆Gbind = binding free energy, Gcomplex = free energy of the complex, Gprotein = free energy of the target protein, and Gligand = free energy of the ligand. The MMGBSA outcome trajectories were analyzed further for post dynamics structure modifications.

### Dynamic Cross Correlation and Principal Component (PCA) Analysis

During a 150 ns MD simulation, a dynamic cross correlation matrix (DCCM) was constructed across all C-atoms for all complexes in order to examine domain correlations. During a 150 ns simulation of ALK and BTK complexed with Wedelosin, PCA analysis was used to recover the global movements of the trajectories. To calculate the PCA, a covariance matrix was created as stated. For conformational analysis of the Wedelosin in bound complex, 10 alternative conformational modes of the main component as movements of trajectories were calculated, and a comparison of the mode PC1, PC2, PC3 and the last modes PC9 and PC10 were investigated to understand the convergence of trajectories. Geo measures v 0.8 was used to calculate the free energy landscape of protein folding on a Wedelosin -bound complex (Kagami et al., 2020). The MD trajectory versus PC2 energy profiles of folding was recorded in a 3D plot using the matplotlib python package using Geo measures, which includes a comprehensive library of g_sham.

## Results

### QSAR

The statistical parameters associated with fitting, double validation and Y-scrambling for *de novo* QSAR model with threshold values for some of the parameters are presented below.

**QSAR Model** (Divided Set: Training Set-80% and Prediction Set-20%):

**Q** ^**2**^_**loo**_ **: 0.8066, R** ^**2**^ **:0.8253**, R^2^_adj_: 0.8184,R^2^-Q^2^_loo_: 0.0188, R^2^-R^2^_adj_: 0.0069,K_xx_: 0.2845, Delta K: 0.0918,RMSE_tr_: 0.6137, RMSE_cv_: 0.6459,RMSE_ex_:0.6959, Sy: 0.6278, **F:118.9309**, **Q** ^**2**^ **-F1: 0.7784, Q** ^**2**^ **-F2: 0.7782, Q** ^**2**^ **-F3: 0.7755**, **CCC**_**tr**_ **:0.9043**, CCC_cv_: 0.8941,CCC_ex_: 0.8919,r^2^m av: 0.7169, r^2^m de: 0.0591,MAE_tr_: 0.4707,MAE_cv_: 0.4944,MAE_ex_: 0.5620,RSS_tr_: 59.5085, PRESS_cv_: 65.9115, PRESS _ex_: 18.8848, **R** ^**2**^_**LMO**_ **:0.828**, **Q** ^**2**^_**LMO**_ **: 0.815**, R^2^_Yscr_: 3.6912,**Q** ^**2**^_**Yscr**_ **: 5.5179**.

**LOO Obs**(x) Pred(y): R^2^:0.8067, R^2^o: 0.7679, K_o_: 0.9920, Clos: 0.0481, Rm: 0.6479.

**LOO Pred**(x) Obs (y): R^2^: 0.8067, R^2^_o_: 0.8066, K_o_: 0.9995, Clos: 0.0002, Rm: 0.7977.

**Calc. Obs(x) Calc(y):**R^2^:0.8000, R^2^o:0.7955, K_o_: 0.9813, Clos: 0.0056, R^2^m:0.7465.

**Calc. Calc (x) Obs(y):**R^2^:0.8000, R^2^o: 0.7801, K_o_: 1.0093, Clos: 0.0248, R^2^m:0.6873.

**(**Threshold Values for some important statistical parameters: R^2^ ≥ 0.6, Q^2^_LOO_ ≥ 0.5, Q^2^_LMO_ ≥ 0.6, R^2^ > Q^2^, R^2^ex ≥ 0.6, RMSE_tr_ < RMSE_cv_, ΔK ≥ 0.05, CCC ≥0.80, Q^2^-F^n^ ≥ 0.60, r^2^_m_ ≥ 0.6, 0.9 ≤ k ≤ 1.1, and 0.9 ≤ k’ ≤ 1.1 with RMSE≈0, MAE ≈0).

**FIGURE 2:**
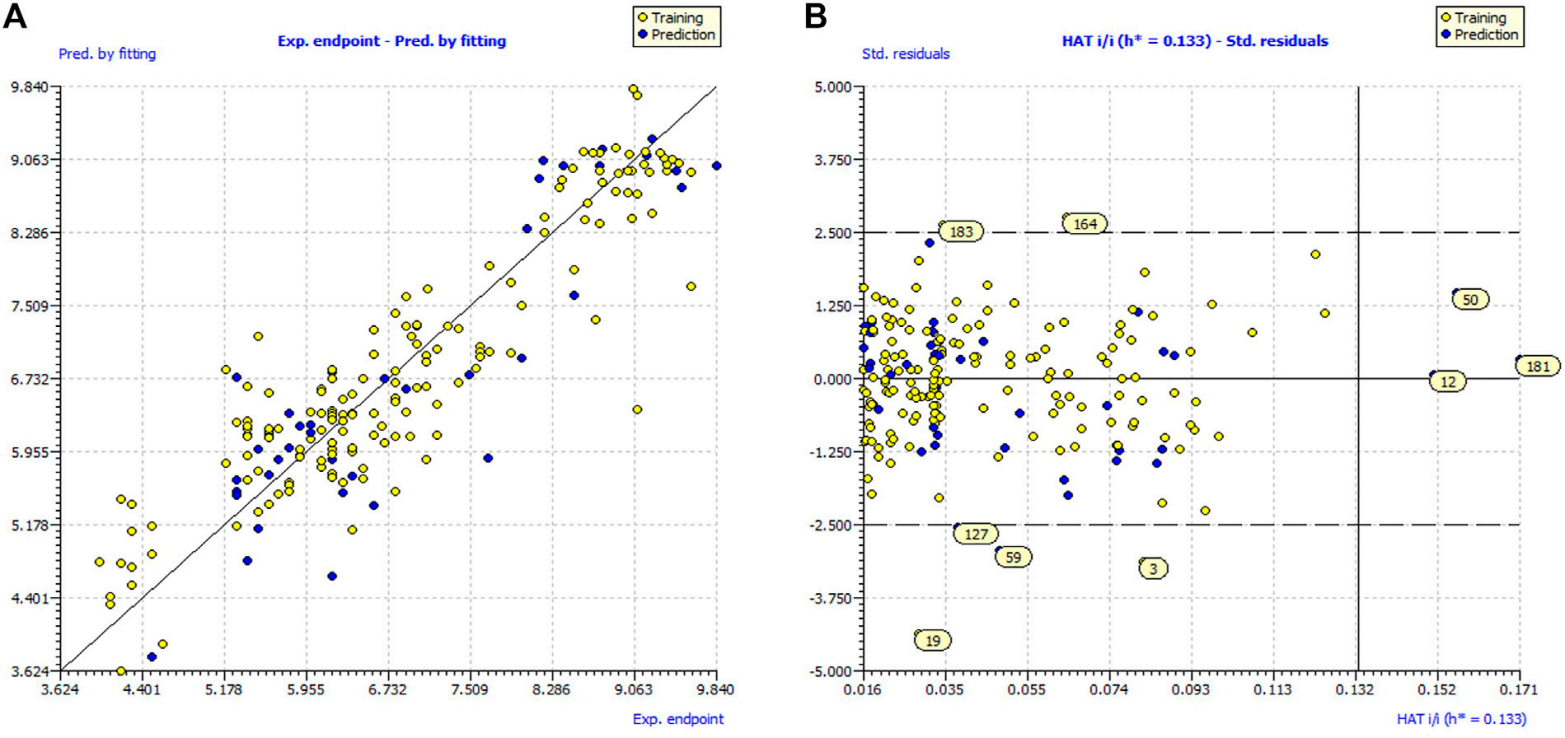
**(A)** Graph of experimental vs. Predicted pIC_50_ values for model; **(B)** Williams plot for model applicability domain of model.

Values of fitting parameters (R^2^, R^2^_adj_ etc.) are well above the approved thresholds which confirm the adequacy of number of molecular descriptors in the models and statistical acceptability of the QSAR models. Values of Q^2^_LOO_, Q^2^_LMO_ etc. (Internal validation parameters) vouchsafed the statistical robustness of the QSAR models. High values of external validation parameters R^2^_ex_, Q^2^F^1^, Q^2^F^2^, Q^2^F^3^ etc., highlights the external predictability of all the three models. Model applicability domain (AD) is validated from Williams plots for model (see Figure 2). Almost all the statistical parameters have attained values well above the approved threshold values and minimal correlation among the selected molecular descriptors ruled out the possibility of chancy QSAR model development (Supplementary Tables S2, S3). These evidences support the models’ statistical robustness and high external predictability.

QSAR Model (Divided Set: Training Set-80% and Prediction Set-20%)

**pKi** = −5.006 (±0) + 0.159 (±0) * **fringNaroC5B**+ 0.459 (±0) *N_hy1 + 0.679 (±0) * **faroNsp2O5B**+ 16.899 (±0) * **rsa** + 0.549 (±0) * **fsp2Oacc6B**+ 0.608 (±0) * **faccsp2C4B**.

### Mechanistic Interpretation of Descriptor

**FIGURE 3:**
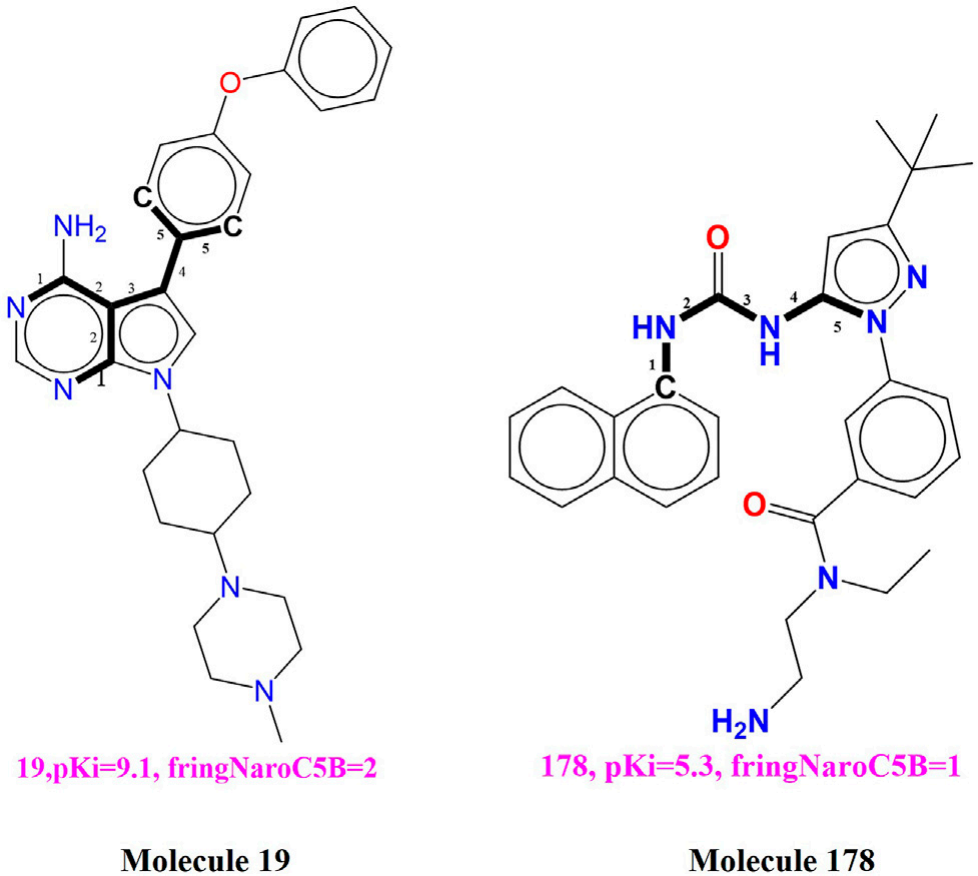
Depiction of the descriptor fringNaroC5B for the molecules 19 and 178 only.

**fringNaroC5B (**Frequency of occurrence of aromatic carbon atoms exactly at five bonds from the ring Nitrogen atoms) This descriptor has acquired positive correlation with the binding coefficient for BTK tyrosine kinase, therefore further increase in the value of the present descriptor may enhances the binding affinity for the BTK tyrosine kinase. This observation is supported by comparing the compound 19 with the compound 178 (see Figure 3). The observation is reinforced by comparing compound **19** (pKi = 9.1) with compound **178** (pKi = 5.3) for which increase in the value of fringNaroC5B from 0 for compound 178 to 2 for compound 178 resulting into increase in the pKi value by about 3.8 unit (about thirty-fold increase in inhibitory potency for BTK tyrosine kinase receptor)**.** Compound **3** (fringNaroC5B = 4; pKi = 9.6) and compound **94** (**f**ringNaroC5B = 1; pKi = 6.7) is one more pair as an example to support this observation.

Furthermore, when we have shifted the molecular descriptor **fringNaroC5B** (**R** ^**2**^ **= 0.82)** with the descriptor **fringNringC5B** (frequency of occurrence of ring carbon atom exactly at five bonds from the ring nitrogen atom). We notified that mere ring carbon atoms are the better choice and crucial for enhancing the binding affinity for BTK tyrosine kinase as portrayed from the statistically significant correlation **(R** ^**2**^ **= 0.85)** with the binding affinity (pKi). Moreover, replacement of aromatic ring carbon atom specifically with the unsaturated ring carbon atom will significantly contribute to enhance the lipophilicity of the molecule. Concurrently, when we have changed the molecular descriptor **fringNaroC5B** with the molecular descriptor **aroC_ringC_5B** (Occurrence of aromatic ring carbon atoms within five bonds from the aromatic carbon atoms) will result into substantial fall in the statistical performance of the developed QSAR model **(R** ^**2**^ **= 0.76).** Therefore, it is settled that distance between aromatic carbon atom and ring nitrogen atom should be five bonds to have better binding affinity for BTK tyrosine kinase.

**FIGURE 4:**
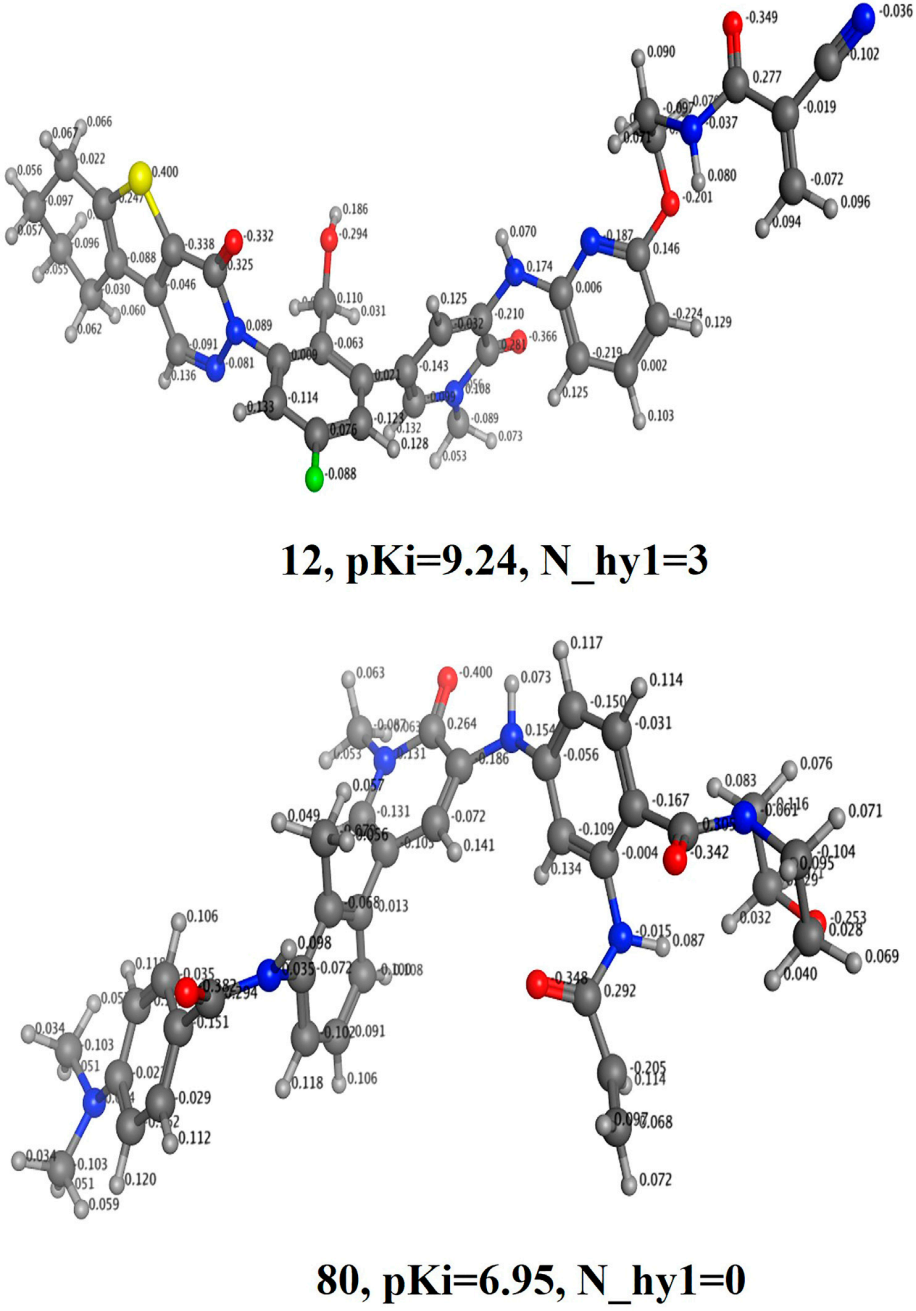
Display of the molecular descriptor N_hy1 for the molecules 12 and 80 only.

**N_hy1 (**Number of nitrogen atoms with the partial charge < −0.100 and > −0.199). The present descriptor shows positive correlation with binding coefficient (Ki) in the developed QSAR model, therefore further increase in the value of this descriptor may plausibly enhances binding affinity against BTK tyrosine kinase receptor. This is observed by comparing the pKi value of the molecule **12**(**N_hy1 = 3; pKi = 9.24**) with the molecule **80** (**N_hy1 = 0; pKi = 6.95**) (see Figure 4). The partial positive/negative charge on the molecule has been enhanced by the addition of variety of polar substituents. For backing the present observation, we have shifted the value of the descriptor **N_hy1 for** the molecule **80** by about 2 that may change the pKi value of the molecule **80** by about 2.29 unit (about twenty-fold increase in the inhibitory potency for BTK tyrosine kinase receptor).

If we shift molecular descriptor **N_hy1** (**R** ^**2**^ **:0.82)** with the descriptor **N_MSA1** (number if nitrogen atoms having partial charge in the range -0.100 to -0.199) will plausibly enhance the statistical significance (**R** ^**2**^ **= 0.87**) of the developed QSAR model. Based on this analysis, it can be recognized that the descriptor **N_MSA1** is the superior option to predict the binding affinity for BTK tyrosine kinase inhibitors. The compound 2 (**N_hy1 =2, pKi = 9.84)** and compound **195** (**N_hy1 = 0, pKi = 4.1**) is one more pair of duos which support this remark.

**FIGURE 5:**
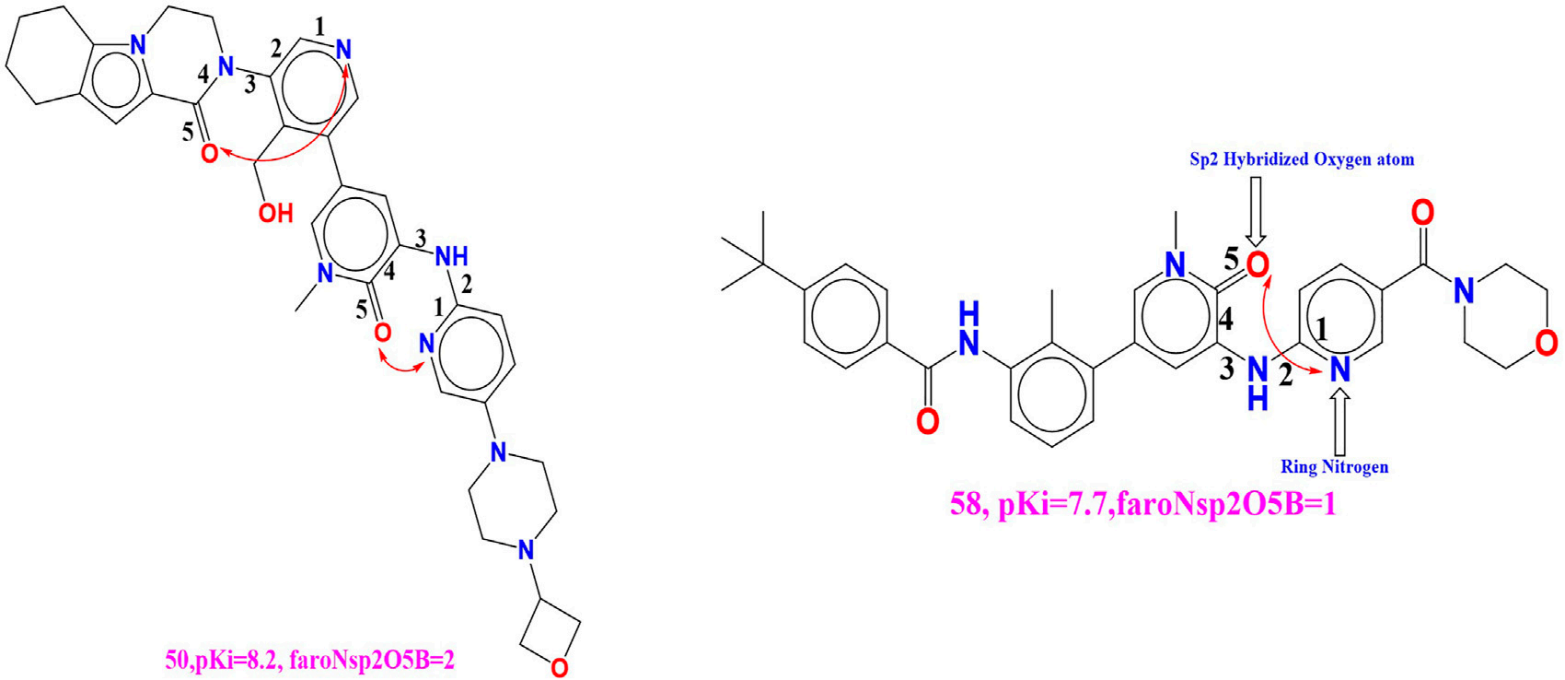
Depiction of the molecular descriptor **faroNsp2O5B** for the molecule 50 and 58 only.

**faroNsp2O5B** (Frequency of occurrence of sp2 hybridized oxygen atoms exactly at 5 bonds from the aromatic nitrogen atoms) The positive numerals for the present descriptor, **faroNsp2O5B** in the created QSAR model will subsequently enhances the binding affinity (Ki) for the BTK tyrosine kinase receptor. We have compared the molecule 50 (**faroNsp2O5B = 2, pKi = 8.2)** with the molecule 58 (**faroNsp2O5B = 2, pKi = 7.7)** to witness the effect of the existing molecular descriptor on binding affinity (Ki) (**see** Figure 5). Compound 4 (**faroNsp2O5B = 1, pKi = 9.51)** and compound 91 (**faroNsp2O5B = 2, pKi = 6.8)** is the additional such pair which reinforce this remark.

**FIGURE 6:**
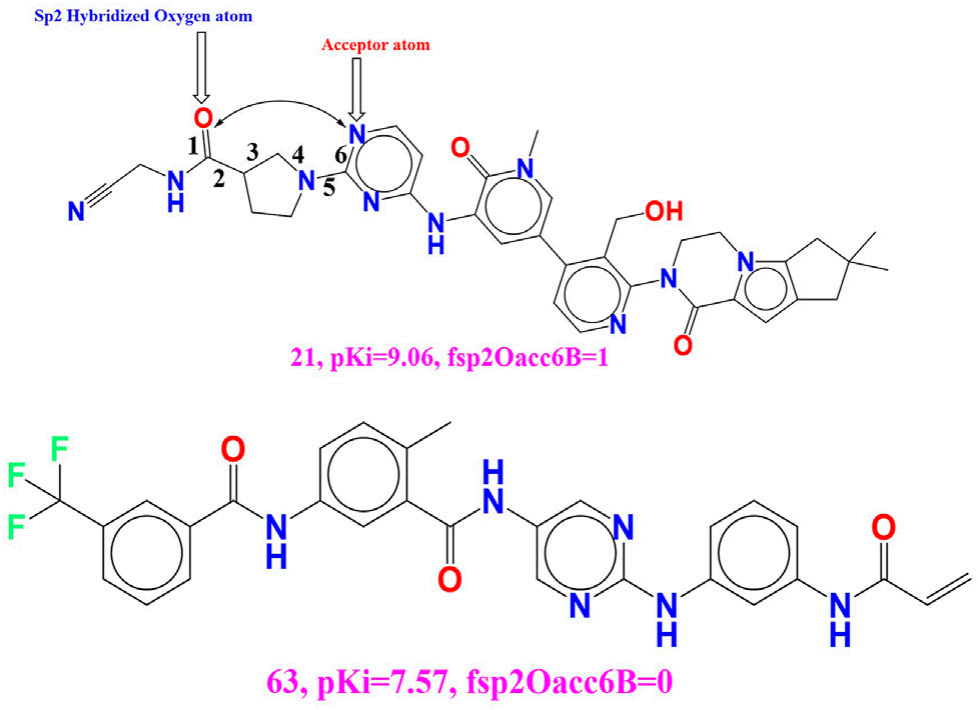
Presentation of the molecular descriptor **fsp2Oacc6B** for the molecule 21 and 63 only.

If we change the value of the molecular descriptor for molecule 58 by one unit, the pKi value will rise by about 0.5 unit (about 5-fold increase in the inhibitory potency for BTK tyrosine kinase receptor). When the molecular descriptor faroNsp2O5B was replaced with the molecular descriptor faroNO5B, the statistical performance (R2 = 0.89) of the created QSAR model was significantly altered (R2 = 0.89) (presence of oxygen atom exactly 5 bonds from the aromatic carbon atom). As a result, while adding an oxygen atom to a BTK tyrosine kinase receptor increases binding affinity, faroNO5B is a better predictor of BTK tyrosine kinase inhibitor binding affinity.

rsa (Ratio of molecular surface area to the solvent accessible surface area). Because these descriptors have a positive coefficient in the established QSAR model, increasing the value of rsa will most likely increase the BTK tyrosine kinase binding affinity. This conclusion is supported by comparing the molecules 52 (pKi = 8.1, rsa = 0.735) and 71 (pKi = 7.1, rsa = 0.696). If we change the value of rsa for molecule 71 to 0.3, the BTK tyrosine kinase inhibitory potency (pKi) increases by approximately 1 unit (about 10-fold increase in inhibitory potency for BTK tyrosine kinase receptor). The developed QSAR model’s performance (R2 = 0.85) is statistically altered by replacing the molecular descriptor rsa with the molecular descriptor notringC MSA (molecular surface is of all non-ring carbon atoms). As a result, the molecular surface area of non-ring carbon atoms must be considered for future lead optimization of BTK tyrosine kinase inhibitors. fsp2Oacc6B (Frequency of occurrence of acceptor atoms exactly at 6 bonds from the sp2 hybridised oxygen atoms) Because this chemical descriptor has a positive correlation with binding affinity (pKi), increasing the descriptor value may increase the pKi value of BTK tyrosine kinase inhibitors. The effect of the present descriptor can be seen by comparing molecules 21 (fsp2Oacc6B = 1; pKi = 9.06) and 63 (fsp2Oacc6B = 0; pKi = 7.57). This finding is supported by comparing the two molecules, 20 (fsp2Oacc6B = 1; pKi = 9.1) and 191 (fsp2Oacc6B = 0; pKi = 4.3) (See Figure 6).

We get a tenfold increase in the inhibitory potency (pKi) for the BTK tyrosine kinase receptor if we increase the pKi value by 1.49 unit by shifting the value of the molecular descriptor by one for the molecular 63.

The statistical performance of the existing QSAR model changes when the chemical descriptor fsp2Oacc6B is replaced with another molecular descriptor fsp3OringN6B (frequency of occurrence of ring nitrogen atom exactly at 6 bonds from the sp3 hybridized oxygen atoms) (R2: 0.88). As a result, with an ideal distance of 6 bonds between the acceptor and oxygen atoms, the sp3 oxygen atom is the better choice for increasing the potency of BTK tyrosine kinase inhibitors.

**faccsp2C4B** (Frequency of occurrence of sp2 hybridized carbon atoms exactly at 4 bonds from the acceptor atoms) This descriptor has attain positive numeral in the developed QSAR model and for the small increase in the value of the present descriptor gave rise to amplification in the binding affinity (Ki) for the BTK tyrosine kinase.

**FIGURE 7:**
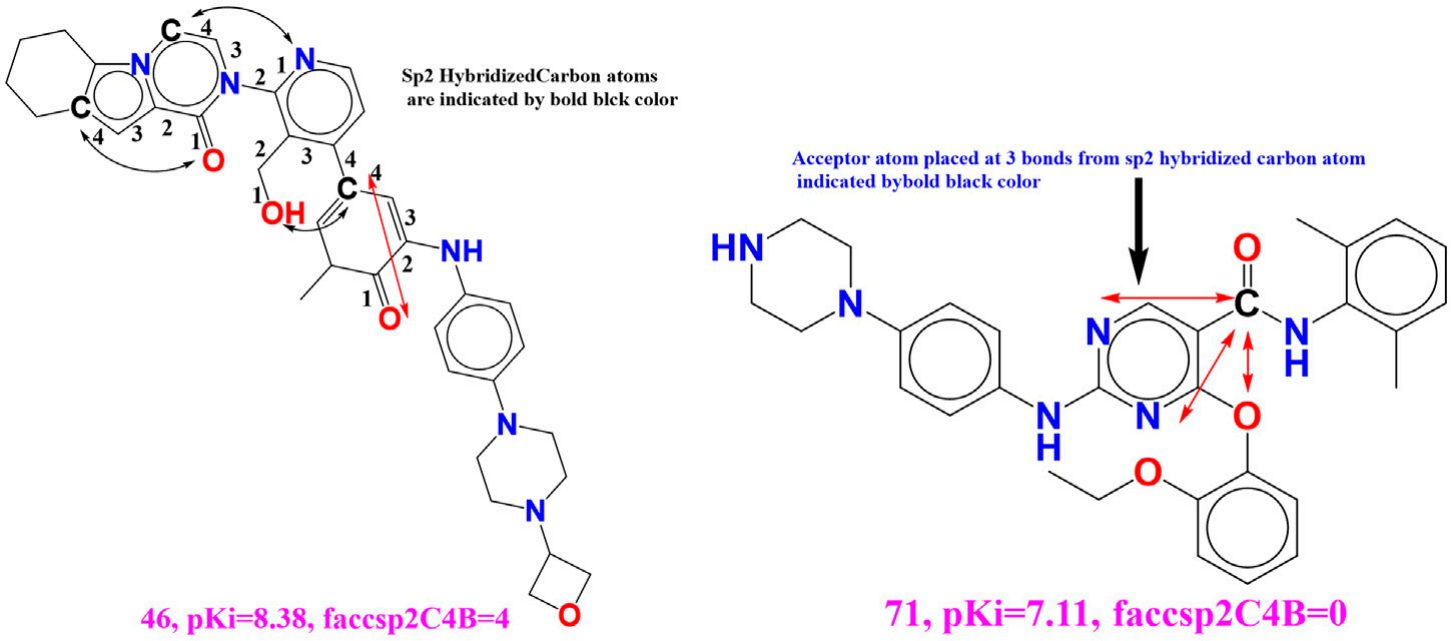
Portrayal of the molecular descriptor faccsp2C4B for the molecular 46 and 71 only.

This can be illustrated by comparing the molecule **46 (faccsp2C4B = 4, pKi = 8.38)** with the molecule **71 (faccsp2C4B = 0, pKi = 7.11)**. (see Figure 7). The basic difference which occurs amid two molecules is sp2 hybridized carbon which seems to be imperative for the enhancing the binding affinity for BTK tyrosine kinase receptor. If we shift the value of the molecular descriptor by 4 for the molecular 71 will results into increase the pKi value by 1.27 unit (about tenfold increase in the inhibitory potency (pKi) for the BTK tyrosine kinase receptor). Moreover, increase in the carbon atom content in the molecule may gave rise increase in the lipophilic character of the molecule, therefore sp2 hybridized carbon atom is important for the future optimization of the lead molecule. Concurrently, when we have shifted the molecular descriptor **faccsp2C4B** with the molecular descriptor **faccsp2C3B** (frequency of occurrence of sp2 hybridized carbon atom exactly at 3 bonds from the acceptor atoms) will significantly drop the statistical performance **(R** ^**2**^ **= 0.75)** of the created QSAR model. Therefore, we can infer that the optimum distance between the acceptor atom and sp2 hybridized carbon atom must be 4 bonds to enhance the binding affinity for the BTK tyrosine kinase receptor. Additionally, the molecular descriptor **faccsp2C4B** is the better selection to predict the binding affinity for BTK tyrosine kinase receptors.

### Structure Based (Dock) Virtual Screening

High throughput structure based virtual screening of 161 olive metabolites were analyzed in dock-based approach against ALK and BTK protein. The binding site residues for specific docking were determined from the native ligand in X-ray crystallography structures associated with the ALK and BTK protein using DogSiteScorer. Moreover, structural superimposition of ALK and BTK with respective co-crystallized ligand and Wedelosin are displayed in Supplementary Figures S1A,B, respectively, for validation of binding sites. In ALK protein the binding site of the native ligand predominated with amino acid residues LEU1122, GLY1123, PHE1127, VAL1130, ALA1148, LEU1196, GLU1197, LEU1198, MET1199, ALA1200, GLY1201, GLY1202, ASP1203, ARG1253, ASN1254, CYS1255, LEU1257, GLY1268, ASP1269 within 5 Å space. Whereas, for BTK the residues at the binding site were determined as ARG97, LEU11, LYS26, ILE95, PHE44, ILE9, ASP43, GLU96, GLU76, LYS27, TYR42, ARG28, PRO75. within 5 Å space. The best molecules from **QSAR based virtual screening**, Wedelosin, Oleanolic acid, Corosolic acid, Ursolic acid, Maslinic acid and Pomolic acid also showed significant binding energies < -8.0 (kcal/mol) when processed in structure-based ligand screening displayed in Table 1.

**TABLE 1:** Binding energies of ligands with ALK and BTK as determined from structure based ligand screening.

Compound	Binding energies with ALK (kcal/mol)	Binding energies with BTK (kcal/mol)
Wedelosin	−8.6	−8.04
Oleanolic acid	−8.4	−7.7
Maslinic acid	−8.3	−7.6
Corosolic acid	−8.2	−7.4
Ursolic acid	−8.4	−7.2
Pomolic acid	−8.2	−7.01

### Molecular Docking for Validation of Docking Score

**FIGURE 8:**
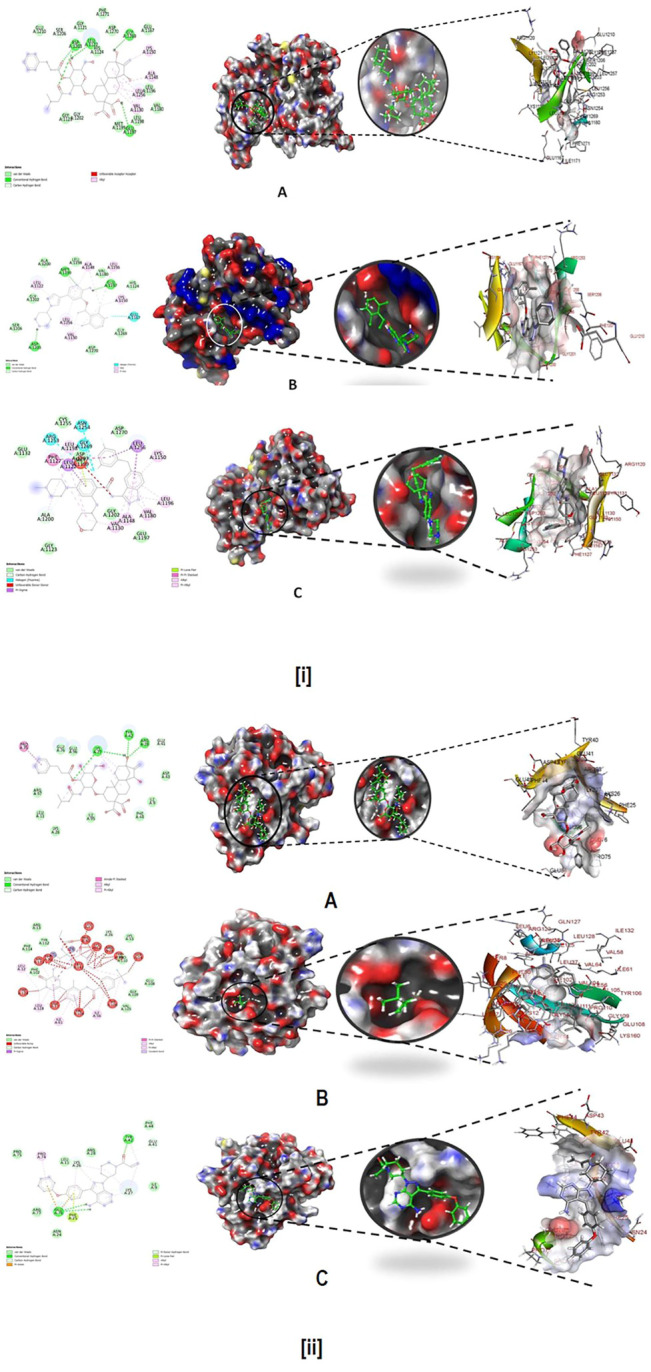
**(i)** Best docked pose of **(A)** Wedelosin, **(B)** entrectinib, **(C)** crizotinib with ALK displaying 2D interaction plot on the left panel. Pink dashed lines indicating the Pi-Alkyl bond and residues embedded in light green sphere indicating to involve in Van der Waals interactions. On the center panel, surface view of ALK displaying binding cavity of Wedelosin and right panel displaying the zoomed out binding pocket having amino acid residues surrounding the Wedelosin molecule; **(ii)** Best docked pose of **(A)** Wedelosin, **(B)** N42, **(C)** ibrutinib with BTK displaying 2D interaction plot on the left panel. Pink dashed lines indicating the Pi-Alkyl bond and residues embedded in light green sphere indicating to involve in Van der Waals interactions. On the center panel, surface view of BTK displaying binding cavity of Wedelosin and right panel displaying the zoomed out binding pocket having amino acid residues surrounding the Wedelosin molecule.

The best molecule Wedelosin from virtual screening having lowest binding energy was subjected to molecular docking with ALK and BTK receptors. In addition to that, docking with co-crystallized ligand, approved drugs showed in Figure 8. All the dock scores displayed to having low RMSD tolerance 0.25 Å and binding energy fall maximum within that RMSD cluster. Free energy of binding of Wedelosin with ALK exhibited (∆G) −8.6 kcal/mol, inhibitory concentration (Ki) 2.55 µM, ligand efficiency −0.26, total internal energy -0.45 kJ/mol, and torsional energy 0.3 kJ/mol. Whereas, the redocked co-crystallized ligand entrectinib exhibited binding energy −8.2 kcal/mol, inhibitory concentration (Ki) 6.75 µM, ligand efficiency −0.16, total internal energy −0.23 kJ/mol, and torsional energy 0.6 kJ/mol. On the other hand, the approved displayed low affinity for the ALK receptor with binding energy −7.6 kcal/mol, inhibitory concentration (Ki) 55.75 µM, ligand efficiency −0.06, total internal energy −0.11 kJ/mol, and torsional energy 1.6 kJ/mol. The principal residues making the binding pocket around Wedelosin is comprised of Glu1210, Gly1121, Phe1271, Asp1270, Glu1167, Leu1196, Val1180, Met1199, Gly1123 by van der Walls interaction force; residues Gly1202, Ser1206 possess Carbon Hydrogen bonding; residues Val1130, Leu1256, Ala1148, Lys1150 undergoes Alkyl bonding; Asp1203, Leu1122, Gly1269, Glu1197 possess Conventional hydrogen bonding (Figure 8iA, left). The RMSD differences were observed in Wedelosin ALK bound complex with co-crystallized ligand and approved drug 0.129 Å and 0.215 Å, respectively. Interactions of ALK with entrectinib and crizotinib are displayed in Figures 8iB,C , respectively. Wedelosin also displayed better binding energy with BTK as compared to approved drug inhibitor ibrutinib and co-crystallized ligand N42. The Wedelosin bound complex exhibited the binding into the cavity of BTK and residues Arg97, Leu11, Lys26, Ile95, Phe44, Ile9, Asp43, Glu96, Glu76 possess van der Walls interaction force; residues Lys27, Tyr42, Arg28 possess Conventional hydrogen bonding; residue Pro75 possess Amide-Pi Stacked bonding which are held with a binding energy of −8.04 kcal/mol, inhibitory concentration (Ki) 7.34 µM, ligand efficiency −0.11, total internal energy −4.57 kJ/mol, and torsional energy 0.67 kJ/mol. (Figure 8iiA, left). On the other hand, with co-crystallized ligand N42 and approved drug ibrutinib, BTK displayed binding energies −7.8 kcal/mol and −7.6 kcal/mol, with inhibitory concentrations (Ki) 22 and 32 µM, respectively. The RMSD differences were observed in Wedelosin BTK bound complex with co-crystallized ligand and approved drug 0.212 Å and 0.243 Å, respectively Interactions of BTK with N42 and ibrutinib are displayed in Figures 8iiB,C , respectively. Therefore, the docking studies it can be suggested that Wedelosin has better predicted binding energy and inhibitory effect on ALK and BTK over the co-crystallized ligand and approved drugs.

### Molecular Dynamics Simulation (MD) and Free Energy Landscape Analysis

**FIGURE 9:**
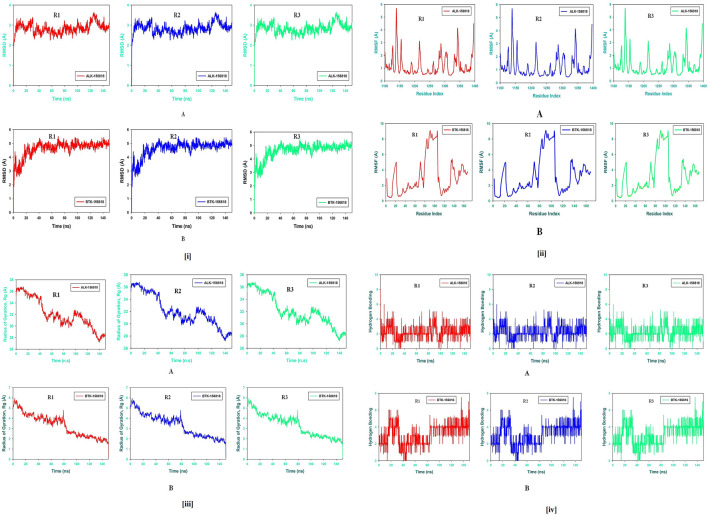
**(i) (A)** MD simulation trajectory analysis of Root Mean Square Divisions (RMSD) of 156,818 (Wedelosin) bound with ALK at 150 ns time frame in triplicate displayed: R1 (replicate 1) H-Bond plot of 156,818 bound ALK (red); R2 (replicate 2) H-Bond plot of 156,818 bound ALK (blue); R3 (replicate 3) H-Bond plot of 156,818 bound ALK (light green); **(B)** MD simulation trajectory analysis of Root Mean Square Fluctuations (RMSF) of 156,818 (Wedelosin) bound with BTK at 150 ns time frame in triplicate displayed: R1 (replicate 1) H-Bond plot of 156,818 bound BTK (red); R2 (replicate 2) H-Bond plot of 156,818 bound BTK (blue); R3 (replicate 3) H-Bond plot of 156,818 bound BTK (light green). **(ii) (A)** MD simulation trajectory analysis of Radius of gyration (Rg) of 156,818 (Wedelosin) bound with ALK at 150 ns time frame in triplicate displayed: R1 (replicate 1) H-Bond plot of 156,818 bound ALK (red); R2 (replicate 2) H-Bond plot of 156,818 bound ALK (blue); R3 (replicate 3) H-Bond plot of 156,818 bound ALK (light green); **(B)** MD simulation trajectory analysis of Radius of gyration (Rg) of 156,818 (Wedelosin) bound with BTK at 150 ns time frame in triplicate displayed: R1 (replicate 1) H-Bond plot of 156,818 bound BTK (red); R2 (replicate 2) H-Bond plot of 156,818 bound BTK (blue); R3 (replicate 3) H-Bond plot of 156,818 bound BTK (light green). **(iii) (A)** MD simulation trajectory analysis of Root Mean Square Fluctuations (RMSF) of 156,818 (Wedelosin) bound with ALK at 150 ns time frame in triplicate displayed: R1 (replicate 1) H-Bond plot of 156,818 bound ALK (red); R2 (replicate 2) H-Bond plot of 156,818 bound ALK (blue); R3 (replicate 3) H-Bond plot of 156,818 bound ALK (light green); **(B)** MD simulation trajectory analysis of Root Mean Square Fluctuations (RMSF) of 156,818 (Wedelosin) bound with BTK at 150 ns time frame in triplicate displayed: R1 (replicate 1) H-Bond plot of 156,818 bound BTK (red); R2 (replicate 2) H-Bond plot of 156,818 bound BTK (blue); R3 (replicate 3) H-Bond plot of 156,818 bound BTK (light green). **(iv) (A)** MD simulation trajectory analysis of Hydrogen Bonding (H-Bonds) of 156,818 (Wedelosin) bound with ALK at 150 ns time frame in triplicate displayed: R1 (replicate 1) H-Bond plot of 156,818 bound ALK (red); R2 (replicate 2) H-Bond plot of 156,818 bound ALK (blue); R3 (replicate 3) H-Bond plot of 156,818 bound ALK (light green). **(iv) (B)** MD simulation trajectory analysis of Hydrogen Bonding (H-Bonds) of 156818 (Wedelosin) bound with BTK at 150 ns time frame in triplicate displayed: R1 (replicate 1) H-Bond plot of 156818 bound BTK (red); R2 (replicate 2) H-Bond plot of 156818 bound BTK (blue); R3 (replicate 3) HBond plot of 156818 bound ALK (light green).

Molecular dynamics and simulation (MD) studies were carried out to determine the stability and convergence of Wedelosin bound ALK and BTK complex. Each simulation of 150 ns displayed stable conformation while comparing the root mean square deviation (RMSD) values. The Cα-backbone of ALK bound to Wedelosin exhibited a deviation of 1.3 Å (Figure 9iA) in BTK bounded with Wedelosin, a fluctuation of 2.1 Å (Figure 9iB) is found. RMSD plots are within the acceptable range signifying the stability of proteins in the Wedelosin bound state before and after simulation and it can also be suggested that Wedelosin bound ALK (**PDB i.d**: 5FTO) and BTK (**PDB i.d: 5J87)** is quite stable in complex might be due to significant binding of the ligand.

Radius of gyration is the measure of the compactness of the protein. In Wedelosin bound proteins displayed lowering of Radius of Gyration (Rg) (Figures 9iiA,B; R1, R2, R3). Lowering of Rg indicating the compactness of the protein ligand complex. From the overall quality analysis from RMSD and Rg, it can be suggested that Wedelosin bound to the protein targets posthumously in the binding cavities and plays a significant role in the stability of the proteins.

The plots for root mean square fluctuations (RMSF) displayed a significant RMSF in ALK and BTK protein at few residues at the specific time function of 150 ns From the triplicate runs of ALK as shown in Figure 9iiiA, a few fluctuating peaks can be seen although mostly the complex is found to be stabilized from Figure 9iiiB, a fluctuation from residue index 81 till 116 can be found but later it got stabilized. While comparing with the docking results it was observed that in ALK-Wedelosin complex, Leu1122, GLU1197 and Asp1203 those involve in conventional hydrogen bonds formation having RMSF 1.5, 1.2 and 0.5 Å (Figure 9iiiA). All these residue fluctuations are less compared to other residues and therefore suggesting the hydrogen bonding facilitate in stabilizing the complex. In BTK Wedelosin bound complex docking results suggested four hydrogen bond formation with residues couple with Lys27, single with Arg28 and Tyr42 and a comparative analysis in MD simulation exhibited 0.2, 0.4 and 1.3 Å, respectively. All these RMSF values are acceptable for stabilizing the protein ligand complex. Therefore, RMSF plots, it can be suggested that the protein structures were stable during simulation in Wedelosin bound conformation.

The average hydrogen bonds formed between Wedelosin and the respective proteins during the 150 ns simulation were also recorded (Figure 9iv)**.** From 0 to 150 ns a formation of hydrogen bonding was found throughout the simulation and same for triplicate MD simulation of Wedelosin with ALK (Figure 9ivA). In Wedelosin-BTK bound complex similarly significant numbers of hydrogen bonds formed (Figure 9ivB). Moreover, pattern of three and four hydrogen bond formation with ALK and BTK, respectively, in docking was corroborated the number of hydrogen plot analysis after 150 ns molecular dynamics (Figures 9ivA,B). The amount of hydrogen bonds between ALK and BTK with Wedelosin have strengthened the binding and facilitating to conform into more stable complex during the simulation.

**FIGURE 10:**
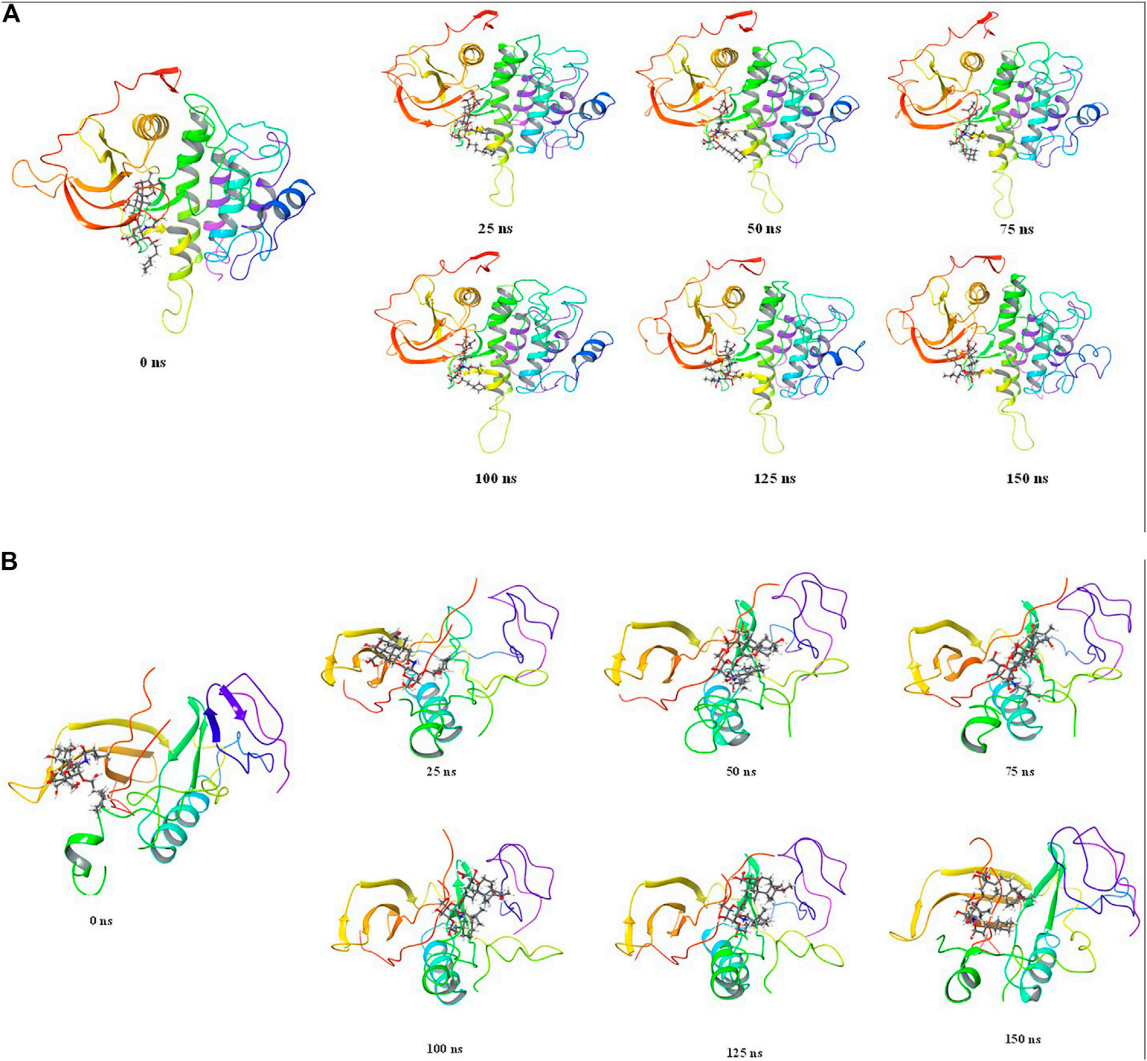
Stepwise trajectory analysis for every 25 ns displaying the protein and ligand conformation during 150 ns of simulation of **(A)**. ALK-Wedelosin and **(B)** BTK-Wedelosin.

The stepwise trajectory analysis of every 25 ns of simulation of Wedelosin with ALK tyrosine kinase displayed the positional alteration with reference to 0 ns structure (Figure 10A). It has been observed that the ligand, Wedelosin have possessed a structural angular movement at the end frame to achieve its conformational stability and convergence. Whereas in case of BTK bounded with Wedelosin possess an angular rotational movement to achieve its structural stability (Figure 10B).

The free energy landscape of (FEL) of achieving global minima of Cα backbone atoms of proteins with respect to RMSD and radius of gyration (Rg) are displayed in Figure 11i. ALK bound to Wedelosin achieved the global minima (lowest free energy state) at 2.8 Å and Rg 33 Å (Figure 11iA). The FEL envisaged for deterministic behaviour of ALK to lowest energy state owing to its high stability and best conformation at Wedelosin bound state. Whereas in case of BTK bound with Wedelosin, the global minima (lowest free energy state) is achieved at 5.3 Å and Rg 16.7 Å (**Figure iB**). Therefore, FEL is the indicator of the protein folding to attain minimum energy state, and that aptly achieved due to Wedelosin bound state.

**FIGURE 11:**
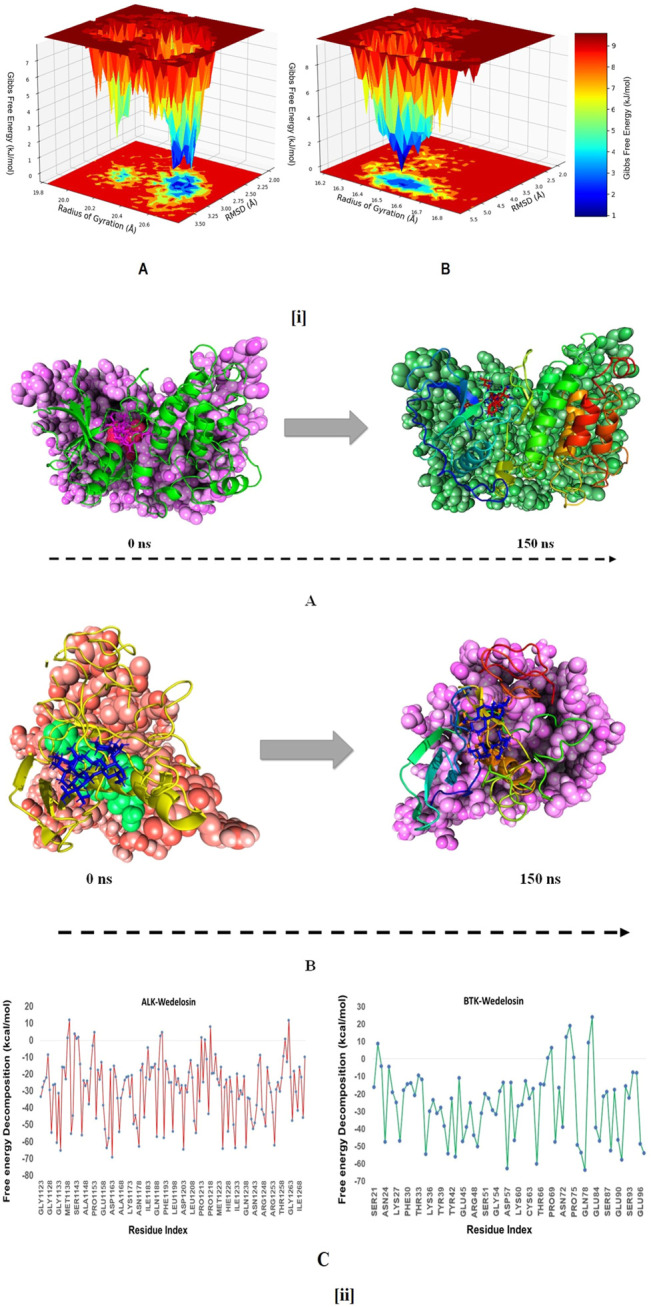
**(i) (A)** Free Energy Landscape displaying the achievement of global minima (ΔG, kJ/mol) of (P) ALK in presence of Wedelosin with respect to their RMSD (nm) and radius of gyration (Rg, nm); **(B)** Free Energy Landscape displaying the achievement of global minima (ΔG, kJ/mol) of (P) BTK in presence of Wedelosin with respect to their RMSD (nm) and radius of gyration (Rg, nm). **(ii)** MMGBSA trajectory (0 ns, before simulation and 150 ns, after simulation) exhibited conformational changes of Wedelosin upon binding with the proteins, **(A)** ALK; **(B)** BTK. The arrows indicating the overall positional variation (movement and pose) of Wedelosin at the binding site cavity; **(C)** Binding free energy decomposition for each residue of the ligands in the complexes (ALK-Wedelosin and BTK-Wedelosin).

**FIGURE 11:**
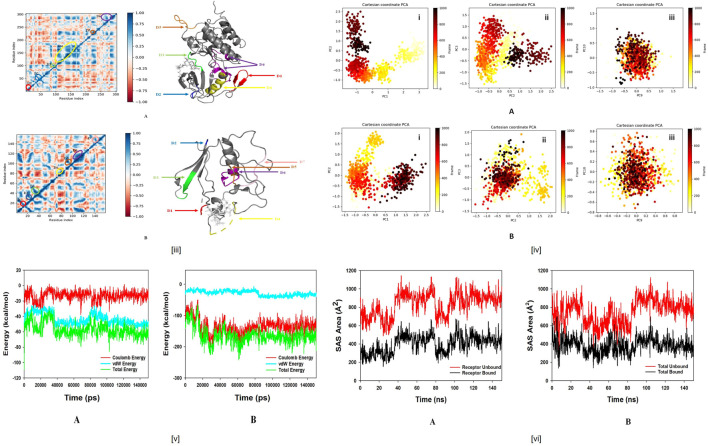
**(iiiA).** Dynamic Cross Correlation matrix (DCCM) of ALK and correlated amino acids conformed into secondary structural domains (coloured) and non-correlated domains (grey) of ALK; **(B)** Dynamic Cross Correlation matrix (DCCM) of BTK and correlated amino acids conformed into secondary structural domains (coloured) and non-correlated domains (grey) of BTK. **(iv)** Principal component analysis (PCA) of **(A).** ALK-Wedelosin displaying **(i)** PC1 and PC2, **(ii)** PC2 and PC3, **(iii)** PC9 and PC10; **(B)** BTK-Wedelosin showing **(i)** PC1 and PC2, **(ii)** PC2 and PC3, **(iii)** PC9 and PC10, for 150 ns simulation trajectories. **(vA)** Energy plot of protein ALK and Wedelosin complex system during the entire simulation event of 150 ns The total energy (green), van der Waal’s energy (cyan) and coulomb energy (red) of the entire system indicating the stability of the individual systems bound to Wedelosin molecule; **(B)** Energy plot of protein BTK and Wedelosin complex system during the entire simulation event of 150 ns The total energy (dark green), van der Waal’s energy (cyan) and coulomb energy (red) of the entire system indicating the stability of the individual systems bound to Wedelosin molecule. **(vi)** Binding Solvent Accessible Surface area (SASA) of bound and unbound state of **(A)** ALK + Wedelosin; **(B)** BTK + Wedelosin. **(iiiA)** Dynamic Cross Correlation matrix (DCCM) of ALK and correlated amino acids conformed into secondary structural domains (coloured) and noncorrelated domains (grey) of ALK; **(B)** Dynamic Cross Correlation matrix (DCCM) of BTK and correlated amino acids conformed into secondary structural domains (coloured) and noncorrelated domains (grey) of BTK. **(iv)** Principal Component Analysis (PCA) of **(A)**. ALK-Wedelosin displaying (i) PC1 and PC2, (ii) PC2 and PC3, (iii) PC9 and PC10; **(B)** BTKWedelosin showing (i) PC1 and PC2, (ii) PC2 and PC3, (iii) PC9 and PC10, for 150 ns simulation trajectories. **(vA)** Energy plot of protein ALK and Wedelosin complex system during the entire simulation event of 150 ns The total energy (green), van der Waal’s energy (cyan) and coulomb energy (red) of the entire system indicating the stability of the individual systems bound to Wedelosin molecule; (B) Energy plot of protein BTK and Wedelosin complex system during the entire simulation event of 150 ns The total energy (dark green), van der Waal’s energy (cyan) and coulomb energy (red) of the entire system indicating the stability of the individual systems bound to Wedelosin molecule. **(vi)** Binding Solvent Accessible Surface area (SASA) of bound and unbound state of **(A)** ALK + Wedelosin; **(B)** BTK + Wedelosin.

### Molecular Mechanics Generalized Born and Surface Area (MMGBSA) Calculations

To assess the binding energy of ligands to protein molecules, the MMGBSA technique is commonly employed. The binding free energy of each protein-Wedelosin complex, as well as the impact of other non-bonded interactions energies, were estimated. With ALK, the ligand Wedelosin has a binding energy of −54.6528 kcal/mol. BTK bound to Wedelosin has an average binding energy of −32.1878 kcal/mol (Table 2). Non-bonded interactions like GbindCoulomb, GbindCovalent, GbindHbond, GbindLipo, GbindSolvGB, and GbindvdW govern Gbind. Across all types of interactions, the GbindvdW, GbindLipo, and GbindCoulomb energies contributed the most to the average binding energy. On the other side, the GbindSolvGB and Gbind Covalent energies contributed the least to the final average binding energies. Furthermore, the GbindHbond interaction values of Wedelosin-protein complexes demonstrated stable hydrogen bonds with amino acid residues. In all of the compounds, GbindSolvGB and GbindCovalent exhibited unfavorable energy contributions, and so opposed binding. Figure 11ii (left panel) reveals that between pre-simulation (0 ns) and post-simulation (0 ns), Wedelosin in the binding pockets of ALK and BTK has undergone a large angular change in the pose (curved to straight) (150 ns). These conformational changes lead to better binding pocket acquisition and interaction with residues, which leads to enhanced stability and binding energy.

**TABLE 2:** Binding energy calculation of Wedelosin with ALK and BTK and non-bonded interaction energies from MMGBSA trajectories.

Energies (kcal/mol) *	ALK	BTK
ΔG_bind_	−54.6528 ± 11.2374	−32.1878 ± 8.5934
ΔG_bind_Lipo	−14.1400 ± 2.5341	−5.61587 ± 2.68045
ΔG_bind_vdW	−46.4764 ± 4.6178	−14.6396 ± 7.62527
ΔG_bind_Coulomb	−22.15048 ± 9.1922	−11.5424 ± 3.0719
ΔG_bind_H_bond_	−2.1925 ± 0.8303	−9.686651 ± 1.85560
ΔG_bind_SolvGB	30.2477 ± 4.7516	29.52873 ± 8.3810
ΔG_bind_Covalent	0.0559 ± 2.0973	9.224784 ± 3.97046

Thus MM-GBSA calculations resulted, from MD simulation trajectories well justified with the binding energy obtained from docking results moreover, the last frame (150 ns) of MMGBSA displayed the positional change of the Wedelosin as compared to 0 ns trajectory signify the better binding pose for best fitting in the binding cavity of the protein (see Figures 11iiA,B).

Therefore, it can be suggested that the Wedelosin molecule has good affinity for the major two targets ALK and BTK. Free energy decomposition of the binding cavity residues of ALK and BTK with Wedelosin were also investigated from MM-GBSA trajectories. In ALK bound Wedelosin displayed the residues LEU1122, GLU1197, ASP1203 and GLY1269 involved in conventional hydrogen bonds as confirmed from molecular docking as well as dynamics studies exhibited very low binding energy decomposition −21.44, −53.83, −64.56 and −21.66 kcal/mol, respectively (Figure 11iiC). However, residues GLY1123, LEU1198, MET1199, HIS1124, GLY1202, ASP1270 involved in Van der Walls and interactions with the ligand contributed significant energies to stabilise the complex (Figure 11iiC). In BTK bound Wedelosin the residues LYS27, ARG28, ASP43, GLU45, ARG48, ARG49 were contributed highest with the ligand binding by involving conventional hydrogen bonding as well as weak non-bonded Van der Waal’s interaction (Figure 11iiC). Therefore, it can be suggested that of the binding site residues of ALK and BTK are principally regulating the stable interaction with the Wedelosin.

### Dynamic Cross Correlation, Principle Component Analysis (PCA), Energy Calculation and Solvent Accessible Surface Area (SASA)

MD simulation trajectories are analyzed for dynamic cross correlation among the domains within protein chains bound with Wedelosin molecule. For correlative dynamic motion, the cross-correlation matrices of ALK and BTK was generated and displayed in Figures 11iiiA,B. The blue blocks displayed in the figure indicated the residues having high correlated movement and red having least correlation. The amino acid residues of Wedelosin bound ALK and Wedelosin bound BTK showed concerted movement of residues (Figure 11iii).

Principal component analysis (PCA) determines the relationship between statistically meaningful conformations (major global motions) sampled during the trajectory. PCA of the MD simulation trajectories for ALK and BTK bound to Wedelosin molecule were analyzed to interpret the randomized global motion of the atoms of amino acid residues. The internal coordinates mobility into three-dimensional space in the spatial time of 150 ns were recorded in a covariance matrix and rational motion of each trajectories are interpreted in the form of orthogonal sets or Eigen vectors. In the ALK and BTK trajectory, PCA indicates the statistically significant conformations. It is possible to identify the major motions within the trajectory as well as the critical motions required for conformational changes. In ALK bound to Wedelosin, two different clusters along the PC1 and PC2 plane have been exhibited that indicating a non-periodic conformational shift **(** Figure 11ivA(i))**.** While, these global motions are periodic because the groupings along the PC2 and PC3 planes do not totally cluster separately (Figure 11ivA(ii))**.** Moreover, high periodic global motion was observed along the PC9 and PC10 planes due to the grouping of trajectories in a single cluster at the center of the PCA plot (Figure 11ivA(iii)). Centring of the trajectories in a single cluster indicates the periodic motion of MD trajectories due to stable conformational global motion. On the other hand, similar behaviour observed in case of PCA analysis of BTK bound to Wedelosin. Here, two different clusters along the PC1 and PC2 plane have been displayed non-periodic conformational shift **(** Figure 11ivB(i)). While, these global motions are periodic because the groupings along the PC2 and PC3 planes do not totally cluster separately **(** Figure 11ivB(ii)). Ordered periodic global motion were observed along the PC9 and PC10 planes due to the grouping of trajectories in a single cluster at the center of the PCA plot (Figure 11ivB(iii)). Centring of the trajectories in a single cluster indicates the periodic motion of MD trajectories due to stable conformational global motion.

The energy profiles of the protein, ALK and Wedelosin complex systems were determined to display the stability of the entire system. In this regard, the total energy (ETOT) of the ALK Wedelosin system shown to be very stable with an average total energy −69.00 kcal/mol (green). However, van der Waal’s energy (vdW) displayed to be merged over the total energy with an average energy −39.00 kcal/mol, contemplating as principal contributor to the stability of the ALK Wedelosin complex (cyan). In addition, Coulombic interactions played minor role in the system stability and contributing an average energy −-23.00 kcal/mol (red), (see Figure11vA). In BTK bounded Wedelosin system, average total energy −130.00 kcal/mol (green) although, van der Waal’s energy (vdW) displayed to be merged over the total energy with an average energy 20.00 kcal/mol, contemplating as principal contributor to the stability of the ALK Wedelosin complex (cyan). In addition, Coulombic interactions played minor role in the system stability and contributing an average energy −101.00 kcal/mol (red), see (Figure 11vB **)**.

Solvent accessible surface area provides the information about the compactness of the protein complex on binding with ligand, here in this case the unbound protein displayed the higher SASA as marked as red as compared Wedelosin bound ALK and BTK which happened due to the compactness of the protein in the bound stage with the ligand as depicted from Figures 11viA,B.

## Discussion

ALK and BTK are the two major targets for inhibition of lung cancer and attraction for the receptor to develop novel drug molecules against the patient suffering from lung cancer as well as SARS-COV-2. Classical 2D-QSAR models link physicochemical properties of substances, such as electronic, hydrophobic, and steric features, to biological activity (Galimberti et al., 2020). In the present communication, we have carried out QSAR modeling studies using 197 diverse set of compounds. The six parameter GA-MLR based QSAR model gave rise to the R2 = 0.8253, Q2LMO: 0.8150, F: 118.93, Q2-F1: 0.7784, Q2-F2: 0.7782, Q2-F3:0.7755, CCCtr: 0.9043, CCCcv: 0.8941 that gives an idea about the pharmacophoric features important for ALK tyrosine and BTK inhibitory activity. As acknowledged earlier, it is vital in conformity with apprehend distinguished and visually no longer recognizable pharmacophoric features related with the inhibitory potency and anticancer activity for ALK and BTK tyrosine kinase for different chemical classes. The QSAR evaluation alongside QSAR primarily based virtual screening have efficaciously recognized the combination concerning reported and novel pharmacophoric features. The QSAR analysis displays that the aromatic carbon atom precisely at a topological distance over 5 bonds from the ring nitrogen but mere ring carbon atom is more beneficial for future lead optimization, wide variety of partially charged nitrogen’s (N_hy1) but nitrogen with partial charge among the length −0.100 after −0.199 (N_MSA1) need to keep included in future drug design, sp2 hybridized oxygen atoms exactly at 5 bonds from the aromatic nitrogen atoms exhibits that the presence about mere oxygen atom alongside aromatic nitrogen is imperative because within future upgradation of lead molecule for anticancer activity. Additionally, some molecular descriptor among QSAR model has investigated the incidence of the carbon atom which in future serve as main site for the optimization over lead molecule. Moreover, certain descriptor has highlighted the incidence of partially charged nitrogen which pointed toward the possible site because of improving the hydrogen bonding interactions against target biomolecule. The QSAR modelling was followed by QSAR based virtual screening strategy to predict the bioactivity of the 161 oleanoic derivatives. From the QSAR based virtual screening study, the best molecule was chosen by using receptor based molecular docking screening that gives novel Wedelosin (pKi = 8.713, K_i_ = 1.94 nM) over other five best known compounds. The structure based virtual screening based on molecular docking also corroborated the findings of QSAR model where Wedelosin displayed lowest binding energy with ALK and BTK receptors. Further re-docking of Wedelosin with both the receptors displayed more binding energy with ALK as compared to BTK. A comparative analysis with the co-crystallized ligand entrectinib and approved drug crizotinib, for ALK receptor Wedelosin displayed similar binding with the former and better than the later. Both entrectinib and crizotinib are popular approved drugs in inhibiting ALK tyrosine kinase for cancer prevention (Awad and Shaw, 2014; Ardini et al., 2016). However, Wedelosin displayed better binding energy with BTK tyrosine kinase as compared to co-crystallized ligands N42 and approved drug ibrutinib with significant predicted inhibitory concentration (Liang et al., 2017). Therefore, from the docking studies it could be predicted that Wedelosin has good affinity for ALK and BTK targets while comparing with approved drugs. MD simulations and binding free energy calculations were performed on chosen protein-ligand complexes based on docking results. RMSD simulations can be used to investigate a protein’s global conformational changes and stability. The root mean square deviation (RMSD) provides a quantitative measure of the similarity of two systems by quantifying the deviations in the proteins’ backbone Cα atoms (Singh et al., 2021). RMSD of ALK and BTK with Wedelosin exhibited a stable conformation having a less deviation representing the formation of a stable conformation with the protein-ligand complex. However, the RMSF displayed very less fluctuation also conforming the stability of the complex as similarly reported elsewhere (Kumar et al., 2014). On the other hand, lowering of radius of gyration depicted the compactness od the ALK and BTK complex with Wedelosin measures the stability of the complex. The formation of the significant number of hydrogen bonds in MD simulation corroborated the findings with molecular docking also suggested for a stable complex formation during the MD simulation over 150 ns time scale. MMGBSA is a powerful tool in determining the binding energy of the ligand with its respective protein targets (Sun et al., 2014). MMGBSA studies accurately predicted the total binding energy of the Wedelosin at the binding cavity of ALK and BTK and exhibited a very low binding energy suggesting the capacity of the Wedelosin to conform into a stable complex. The binding energies in MMGBSA trajectory supported by van der Walls energy, Lipophilic energy, Coulombic energies and similarly reported elsewhere (Bharadwaj et al., 2021). Further free energy landscape (FEL) of Wedelosin bound ALK and BTK complexes exhibited a deep basin over areas of increased free energy with the deep blue colour locations represented the local energy minima and actively promoted stable conformations similarly suggested by Singh et al., 2021. Moreover, in PCA analysis, the trajectories of Wedelosin bound with ALK and BTK in first two PC modes exhibited a less periodic conformation of the global motion whilst later on became congruent due to high conformational stability of the complexes. Therefore, from the overall approach have led to the identification od novel Wedelosin compound from olive fruit which possibly find a new arena of small molecule drug discovery against SARS COV-2 in conjunction with cancer.

## Data Availability

The original contributions presented in the study are included in the article/Supplementary Material, further inquiries can be directed to the corresponding authors.
