# Supplementary material for: Target Specific Inhibition of Protein Tyrosine Kinase in Conjunction With Cancer and SARS-COV-2 by Olive Nutraceuticals
Source: Front Pharmacol. 2022 Mar 8;12:812565. doi: 10.3389/fphar.2021.812565 (PMC8959131; doi:10.3389/fphar.2021.812565)
Supplement: Supplementary file 2 [file Table2.docx]

. Description of the performance parameters in QSARINS

| Performance  parameter | Calculated  during^a^ | Formula^b^ | Description |
| --- | --- | --- | --- |
| *R^2^, R^2^*  *ext* | training, external  validation | ∑^𝑛^ (𝑦_𝑖_ − 𝑦̂_𝑖_)^2^ 𝑅𝑆𝑆  𝑅^2^ = 1 − ^𝑖=1^ = 1 −  ∑^𝑛^ (𝑦 − 𝑦̅)^2^ 𝑇𝑆𝑆  𝑖=1 𝑖 | Explained variance; coefficient of determination, square of the multiple correlation coefficient |
| *R2adj.* | training | 𝑅^2^ = 𝑅^2^ − (1 − 𝑅^2^) × 𝑝  ^𝑎𝑑𝑗.^ 𝑛 − 𝑝 − 1 | R^2^ corrected with the degree of freedom |
| *R^2^ – R^2^*  *adj*. | training | see above | Difference of the two |
| LOF | training | 𝑅𝑆𝑆  𝐿𝑂𝐹 = 2  𝑀 + 𝑑(𝑀 − 1)⁄  l 2  𝑛 I1 − 𝑛 I  𝗁 ) | Friedman lack of fit criteria [40]. M: total number of linearly independent bases in the model, d: degrees-of- freedom cost for each nonlinear basis function |
| *K_x_* | training | Based on PCA, see [41] for details | Inter-correlation among descriptors |
| *ΔK* | training | Based on PCA, see [41] for details | Difference of correlation among descriptors (K_x_) and the |

|  |  |  | descriptors plus responses (K_xy_) |
| --- | --- | --- | --- |
| *RMSE* | training, int. val., ext. val. | ∑^𝑛^ (𝑦 − 𝑦̂ )^2^  𝑅𝑀𝑆𝐸 = √ 𝑖=1 𝑖 𝑖  𝑛 | Root mean square error |
| *MAE* | training, int.  val., ext. val. | ∑^𝑛^ \|𝑦_𝑖_ − 𝑦̂_𝑖_\|  𝑀𝐴𝐸 = ^𝑖=1^  𝑛 | Mean absolute error |
| *RSS* | training | 𝑛  𝑅𝑆𝑆 = ∑(𝑦_𝑖_ − 𝑦̂_𝑖_)^2^  𝑖=1 | Residual sum of squares |
| *CCC* | training, int. val., ext. val. | 2 ∑^𝑛^ (𝑦_𝑖_ − 𝑦̅)(𝑦̂_𝑖_ − 𝑦̂)  𝐶𝐶𝐶 = ^𝑖=1^  ∑^𝑛^ (𝑦_𝑖_ − 𝑦̅)^2^ + ∑^𝑛^ (𝑦̂_𝑖_ − 𝑦̂)^2^ + 𝑛(𝑦̅ − 𝑦̂)^2^  𝑖=1 𝑖=1 | Coefficient of concordance, concordance correlation coefficient [42,43] |
| *s* | training | 𝑁  1  𝑠 = √ ∑(𝑦_𝑖_ − 𝑦̂_𝑖_)^2^  𝑁 − 1  𝑖=1 | Standard error of the estimate |
| *F* | training | ∑ 𝑁 (𝑦̅ − 𝑦̂_𝑖_)^2^ ∑^𝑁^ (𝑦_𝑖_ − 𝑦̂_𝑖_)^2^  𝐹 = ( ^𝑖=1^ ) / ( ^𝑖=1^ )  𝑝 − 1 𝑛 − 𝑝 | Fisher value |

| *Q2LOO* | internal  validation | ∑^𝑛^ (𝑦 − 𝑦̂ )2 𝑃𝑅𝐸𝑆𝑆  𝑄2 = 1 − 𝑖=1 𝑖 𝑖/𝑖 = 1 −  ^𝐿𝑂𝑂^ ∑^𝑛^ (𝑦_𝑖_ − 𝑦̅)^2^ 𝑇𝑆𝑆  𝑖=1 | Leave-one-out cross-validated square of the (multiple)  correlation coefficient |
| --- | --- | --- | --- |
| *R^2^ – Q^2^_LOO_* | internal  validation | see above | Difference of the two |
| *PRESS* | internal,  external validation | 𝑛  𝑃𝑅𝐸𝑆𝑆 = ∑(𝑦_𝑖_ − 𝑦̂_𝑖/𝑖_)^2^  𝑖=1 | Predicted residual sum of squares (either cross-validated or calculated on the external set) |
| *Q2LMO* | internal validation | ∑^𝑚^ ∑^𝑛^ (𝑦 − 𝑦̂ )2  𝑄2 = 1 − 𝑗=1 𝑖=1 𝑖 𝑖/𝑗  ^𝐿𝑀𝑂^ ∑^𝑛^ (𝑦_𝑖_ − 𝑦̅)^2^  𝑖=1 | Leave-many-out cross-validated square of the (multiple)  correlation coefficient |
| *R2Y-SCRAMBLE* | internal  validation | see above | *R*^2^ of the training set with Y-scrambling [44] |
| *RMSEAvg, Y- SCRAMBLE* | internal  validation | see above | Average RMSE with Y-scrambling [44] |
| *Q2Y-SCRAMBLE* | internal  validation | see above | *Q*^2^_LOO_ of the training set with Y-scrambling [44] |

| *R2RND-DESCR* | internal  validation | see above | *R*^2^ of the training set with randomized descriptors [44] |
| --- | --- | --- | --- |
| *Q2RND-DESCR* | internal  validation | see above | *Q*^2^_LOO_ of the training set with randomized descriptors [44] |
| *R2RND-RESP* | internal  validation | see above | *R*^2^ of the training set with randomized responses [44] |
| *Q2RND-RESP* | internal  validation | see above | *Q*^2^_LOO_ of the training set with randomized responses [44] |
| *Q2F1* | external  validation | ∑^𝑛𝐸𝑋𝑇^(𝑦 − 𝑦̂ )^2^  𝑄2 = 1 − 𝑖=1 𝑖 𝑖  ^𝐹1^ ∑^𝑛𝐸𝑋𝑇^(𝑦 − 𝑦̅ )^2^  𝑖=1 𝑖 𝑇𝑅 | Definition 1 in [45] for *Q*^2^ of the external test set [46],  TR: training set, EXT: external test set |
| *Q2F2* | external  validation | ∑^𝑛𝐸𝑋𝑇^(𝑦 − 𝑦̂ )^2^  𝑄2 = 1 − 𝑖=1 𝑖 𝑖  ^𝐹2^ ∑^𝑛𝐸𝑋𝑇^(𝑦 − 𝑦̅ )^2^  𝑖=1 𝑖 𝐸𝑋𝑇 | Definition 2 in [45] for *Q*^2^ of the external test set [47],  EXT: external test set |
| *Q2F3* | external  validation | ∑^𝑛𝐸𝑋𝑇^(𝑦 − 𝑦̂ )^2^ /𝑛  𝑄2 = 1 − 𝑖=1 𝑖 𝑖 𝐸𝑋𝑇  ^𝐹3^ ∑^𝑛𝑇𝑅^(𝑦 − 𝑦̅ )^2^ /𝑛  𝑖=1 𝑖 𝑇𝑅 𝑇𝑅 | Definition 3 in [45] for *Q*^2^ of the external test set [48],  TR: training set, EXT: external test set |

| *r2m* | external validation | 𝑟^2^ + 𝑟′^2^  𝑟̅̅2̅ = 𝑚 𝑚  𝑚 2 | Here, 𝑟^2^ = 𝑅^2^ × (1 − √𝑅^2^ − 𝑅^2^), where R_0_^2^ is the  𝑚 0  squared correlation coefficient without intercept. 𝑟′^2^ is  𝑚  the same as 𝑟^2^, with the *x* and *y* axes exchanged. [49,50]  𝑚 |
| --- | --- | --- | --- |
| *Δr^2^_m_* | external  validation | ∆𝑟^2^ = 𝑟^2^ − 𝑟′^2^  𝑚 𝑚 𝑚 | See above. |

for *Case study 2* (c.f. figure 10)
