# Supplementary figures and images for: Target Specific Inhibition of Protein Tyrosine Kinase in Conjunction With Cancer and SARS-COV-2 by Olive Nutraceuticals
Source: Front Pharmacol. 2022 Mar 8;12:812565. doi: 10.3389/fphar.2021.812565 (PMC8959131; doi:10.3389/fphar.2021.812565)

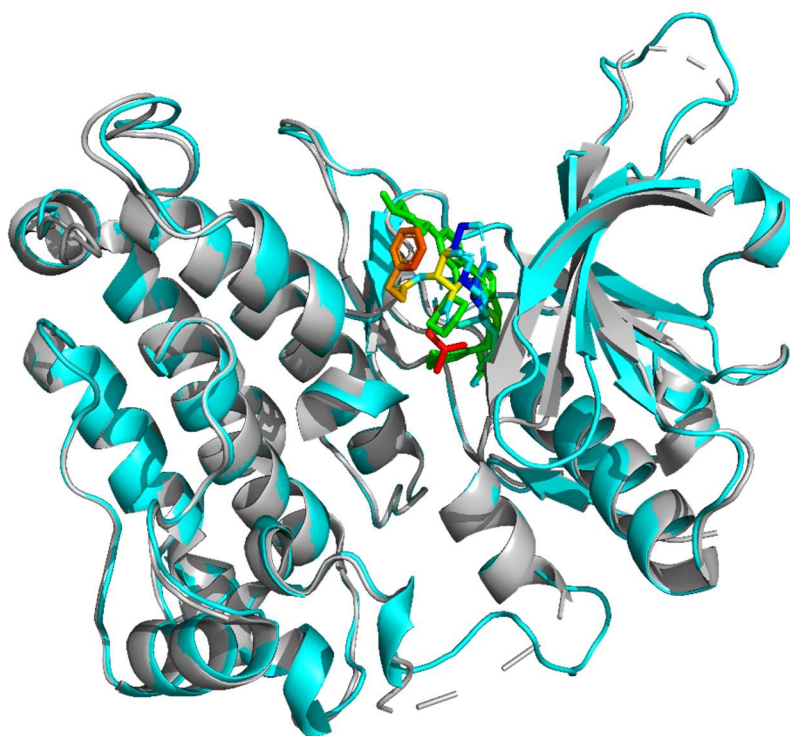

A

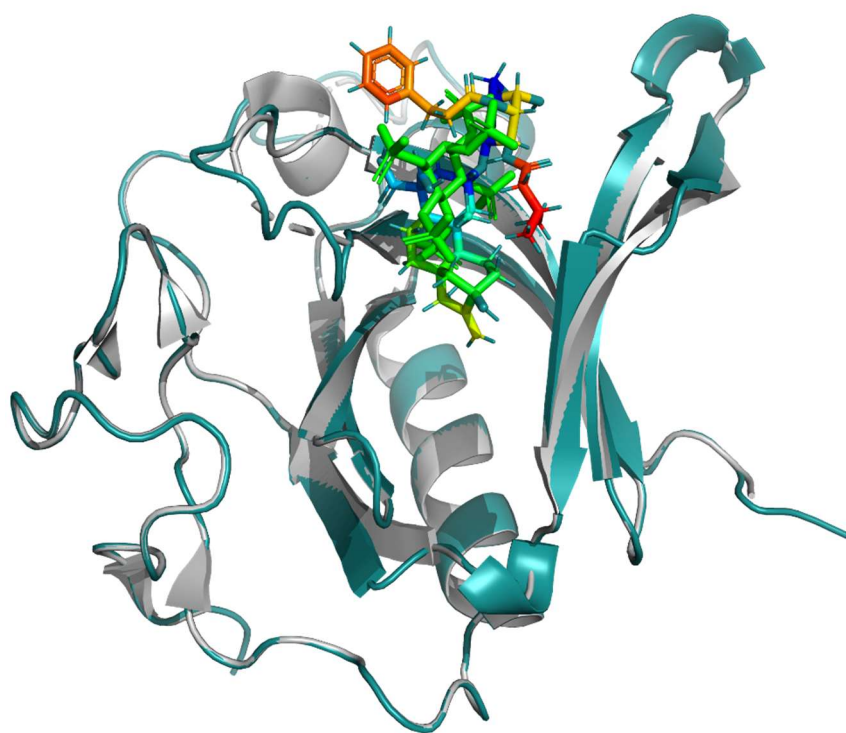

B

Supplement: Supplementary file 5 [file Image1.pdf]
